# Photoinitiated Single-Crystal to Single-Crystal Redox Transformations of Titanium-Oxo Clusters

**DOI:** 10.1021/jacs.4c04068

**Published:** 2024-06-12

**Authors:** Stephen E. Brown, Mark R. Warren, Dominik J. Kubicki, Ann Fitzpatrick, Sebastian D. Pike

**Affiliations:** † Department of Chemistry, University of Warwick, Coventry CV4 7AL, U.K.; ‡ Diamond Light Source, Harwell Science & Innovation Campus, Didcot OX11 0DE, U.K.; § School of Chemistry, University of Birmingham, Birmingham B15 2TT, U.K.; ∥ RAL Space, Harwell Science & Innovation Campus, Didcot OX11 0QX, U.K.

## Abstract

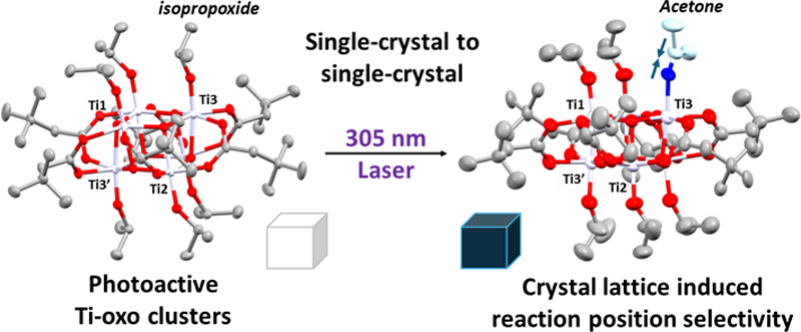

Titanium-oxo clusters can undergo photochemical reactions under UV light, resulting in the reduction of the titanium-oxo core and oxidation of surface ligands. This is an important step in photocatalytic processes in light-absorbing Ti/O-based clusters, metal–organic frameworks, and (nano)material surfaces; however, studying the direct outcome of this photochemical process is challenging due to the fragility of the immediate photoproducts. In this report, titanium-oxo clusters [TiO(O^i^Pr)(L)]_*n*_ (*n* = 4, L = O_2_PPh_2_, or *n* = 6, L = O_2_CCH_2_^t^Bu) undergo a two-electron photoredox reaction in the single-crystal state via an irreversible single-crystal to single-crystal (SC-SC) transformation initiated by a UV laser. The process is monitored by single crystal X-ray diffraction revealing the photoreduction of the cluster with coproduction of an (oxidized) acetone ligand, which is retained in the structure as a ligand to Ti(3+). The results demonstrate that photochemistry of inorganic molecules can be studied in the single crystal phase, allowing characterization of photoproducts which are unstable in the solution phase.

## Introduction

The development of new sustainable (photo)catalytic technologies using earth-abundant metals is a key challenge. As the ninth most abundant element in the Earth’s crust (and the second most abundant transition metal, after iron), titanium offers a great opportunity in this endeavor.^1^ The lighter transition metals typically prefer one electron redox processes, e.g., Ti(4+)/Ti(3+), typically resulting in free-radical based catalytic processes. To perform the multielectron transfer processes associated with selective bond-breaking/making steps commonly needed in catalysis, molecules with multiple redox active metal centers, e.g., titanium-oxo clusters, working together to achieve overall multielectron redox processes is an attractive strategy. In titanium-oxo clusters, light can act as a sustainable energy source to generate highly reactive Ti(3+) sites, which are strongly reducing, along with strongly oxidizing oxygen-based holes. It is important to carefully interrogate the photoredox reactivity of these systems to understand their potential as multielectron redox agents. In this report, in order to study photoproducts directly, without the opportunity for ligand rearrangement, photochemical reactivity is studied in the single-crystalline phase directly by diffraction techniques.

Titanium-oxo clusters have been extensively studied in recent years,^2,3^ finding applications in photocatalysis, photochromism, and as precursors to, and molecular models for, titania-based materials.^4−10^ These clusters may act as the secondary building units in photoactive Ti-based metal organic frameworks (MOFs),^10−17^ which provides further inspiration for study of their photochemistry and reactivity.^18,19^ Further to the assembly of linked 2D or 3D framework materials, there is also interest in directly crystallizing cluster species into functional crystalline materials, which may exhibit reversible sorption properties.^20,21^ These concepts engage with the growing field of solid-state molecular inorganic chemistry,^22−24^ which utilizes the stability and selectivity induced by a crystalline environment to enhance the properties of a molecular species, e.g., for selective catalysis.^25 −27^ When studying molecular chemistry in the crystalline state, an opportunity arises to follow reactivity via single-crystal to single-crystal (SC-SC) transformations.^18,19,28^ If the size and molecular packing of the starting compound and product are similar, crystallinity can be maintained throughout a transformation. This allows the changing molecular structure to be monitored directly using in situ single-crystal diffraction techniques.^29^ SC-SC studies have allowed for the characterization of reactive species which would otherwise be inaccessible or unstable by solution routes.^22,30−39^ Furthermore, extra levels of reaction site selectivity may be imposed by the crystal lattice of a crystalline system. For example, Ozerov reported a Rh-pincer complex which crystallizes with two crystallographically independent molecules; however, only one of these undergoes an intramolecular oxidative addition process in the crystal phase. This indicates that the activation barrier and reaction profile is different in the two different lattice locations, revealing the importance of solid-state interactions to promote or block reactivity.^40−42^ Crystals of metal-oxo clusters have been reported to undergo SC-SC transformations, with examples including oxidation under air and rearrangement and loss of surface ligands.^18,43^

SC-SC transformations may be initiated by exposure to light,^44,45^ for example, the photocyclisation of olefins in the solid-state is well studied.^41,42,46−49^ Irreversible SC-SC transformations induced by photoactivation of inorganic molecules have also yielded fascinating results,^39,50^ with significant recent interest in the photolysis of metal azide or diazoolefin compounds, resulting in the release of N_2_ and formation of intriguing metal nitride/nitrene or metal alkenylidene species.^22,32−38^ In some crystalline materials, photoactivation leads to major geometric changes (i.e., cis–trans isomerization of an azobenzene linkage), causing significant unit cell dimension changes such that crystals (photomechanically) stretch and bend as light falls on their surface. These processes typically occur via amorphization followed by recrystallization of the compound.^51,52^ Furthermore, cocrystals assembled with volatile linkers can be disassembled by low power light, allowing for precise laser cutting of the crystal.^53^ Ito et al. reported crystals which are also observed to jump under UV light (the photosalient effect), caused by shortening of aurophilic intermolecular distances under irradiation.^54^ The development of “photocrystallography,” pioneered by the groups of Coppens and Raithby, also allows interrogation of short-lived photoactivated states by time-resolved crystallography techniques.^55−58^

**Figure 1:**
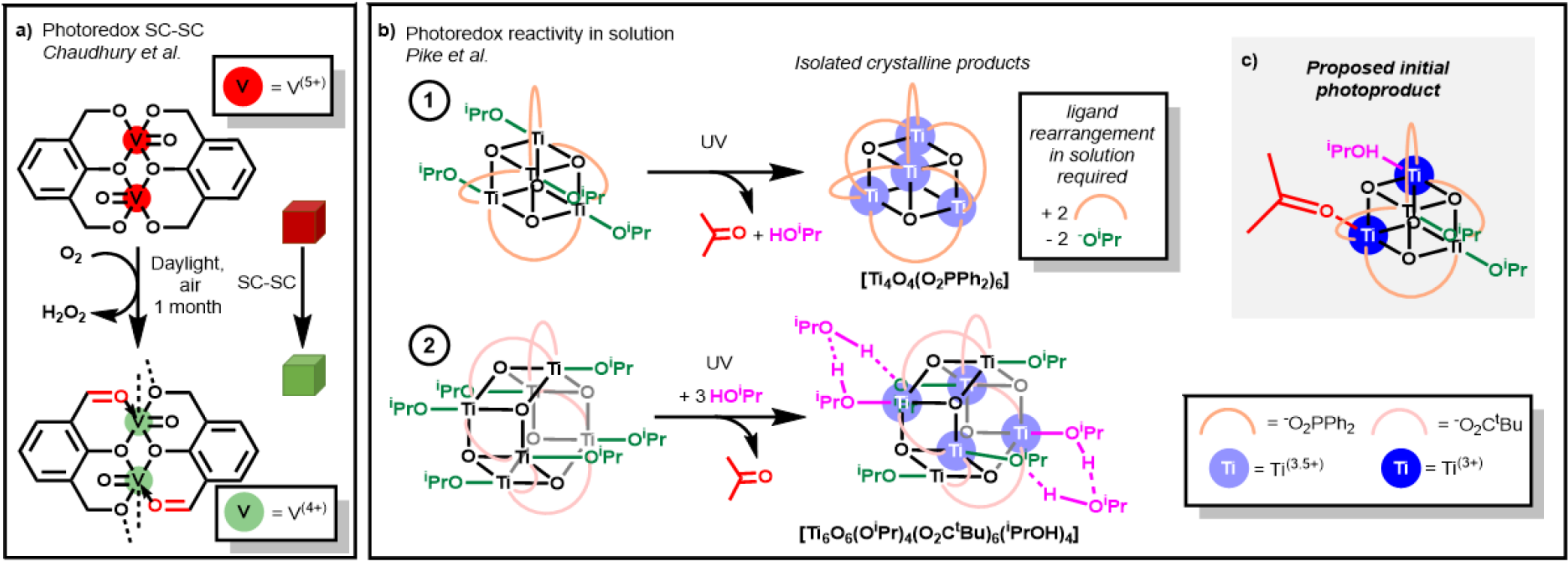
a) Slow photoredox SC-SC transformation of [V_2_O_2_(L)_2_] (H_3_L = 2,6-bis(hydroxymethyl)-p-cresol) under light and air.^59^ b) Photoredox reactivity of **1** and **2** in solution displaying the final isolated products after ligand rearrangements.^18,19^ c) Proposed initial photoproduct of **1** directly after photoredox reaction.

Perhaps the most relevant photoactivated SC-SC example to this study is the slow photoredox reaction of [V_2_O_2_(L)_2_] (H_3_L = 2,6-bis(hydroxymethyl)-p-cresol) under white light and air, resulting in the reduction of the V sites from 5+ to 4+ oxidation state, with corresponding oxidation of the ligand by transforming an alkoxide group to an aldehyde (Figure 1a).^59^

Our research team have studied the photoredox reactivity of Ti-oxo clusters with formula [TiO(O^i^Pr)L]_*n*_ (**1**, L = O_2_PPh_2_, *n* = 4; **2**, L = O_2_C^t^Bu, *n* = 6) in solution under UV light (Figure 1b).^18,19^ These studies reveal that a photoredox process occurs, initiated by an oxygen to metal charge transfer and ultimately resulting in a two-electron-reduced cluster, one molecule of acetone, and one of ^i^PrOH. This photoredox process can be the first step in the photocatalytic oxidation of alcohols under air and UV light. Switching to a cluster with only nonoxidizable O^t^Bu alkoxides significantly slows productive photochemistry,^18^ while photoactivation of the related clusters [Ti_4_O_4_(O^i^Pr)(O^t^Bu)_3_(O_2_PPh_2_)_4_] and [TiO(O(CH_2_)_3_CH=CH_2_)(O_2_C^t^Bu)]_6_ generate exclusively single equivalents of acetone + ^t^BuOH and 4-pentene-1-ol + pent-4-enal, respectively, providing evidence of a dominant two-electron process, which occurs without generation of long-lived organo-free-radicals.^18,19^ Coordinating solvent molecules significantly accelerate the rate of photoreaction and become bound to the Ti^3+^ sites generated by displacing weakly bound ketone and alcohol ligands formed in the photoredox process. The previously reported solution phase studies reveal structural information on the photoproducts only after ligand exchanges have occurred (Figure 1b) but cannot capture the initial photoproduct, including the organic photoproducts while they are still coordinated to the surface (a suggested structure for the directly formed photoproduct is shown in Figure 1c). In particular, acetone is an easily displaced weakly coordinating neutral ligand. In this study, the immediate photoproducts are trapped within a single-crystalline environment and can be directly characterized using single-crystal diffraction using a bespoke laser irradiation, inert gas protection, and synchrotron X-ray diffraction experimental setup. Solid-state NMR, UV–vis, and EPR spectroscopies are used to support the crystallographic findings, confirming that the two-electron photoredox process occurs in the solid state.

## Results and Discussion

Compounds **1** and **2** were prepared following the literature procedures,^18,19^ which comprise the 1:1:1 reaction of Ti(O^i^Pr)_4_, acidic proligand and water in toluene at 60 °C. **1** typically cocrystallizes with one solvent molecule per cluster, allowing for a range of different crystalline forms to be prepared with differing solvents present in the crystal lattice. A range of these crystal forms were explored for solid-state photochemistry and gave broadly similar results (see, Table S1 and Supporting Note 1). Considering that pyridine (py) is known to act as a good solvent to coordinate to photoreduced clusters, **1.py** was chosen as an interesting system for solid-state photochemical study.^18,19^ [Ti_6_O_6_(O^i^Pr)_6_(O_2_CCH_2_^t^Bu)_6_], **3**,^60^ was prepared in a similar manner to structurally analogous **2**.^19^**3** exhibits less structural disorder and polymorphism than **2**, making it a better candidate for SC-SC studies.

**Figure 2:**
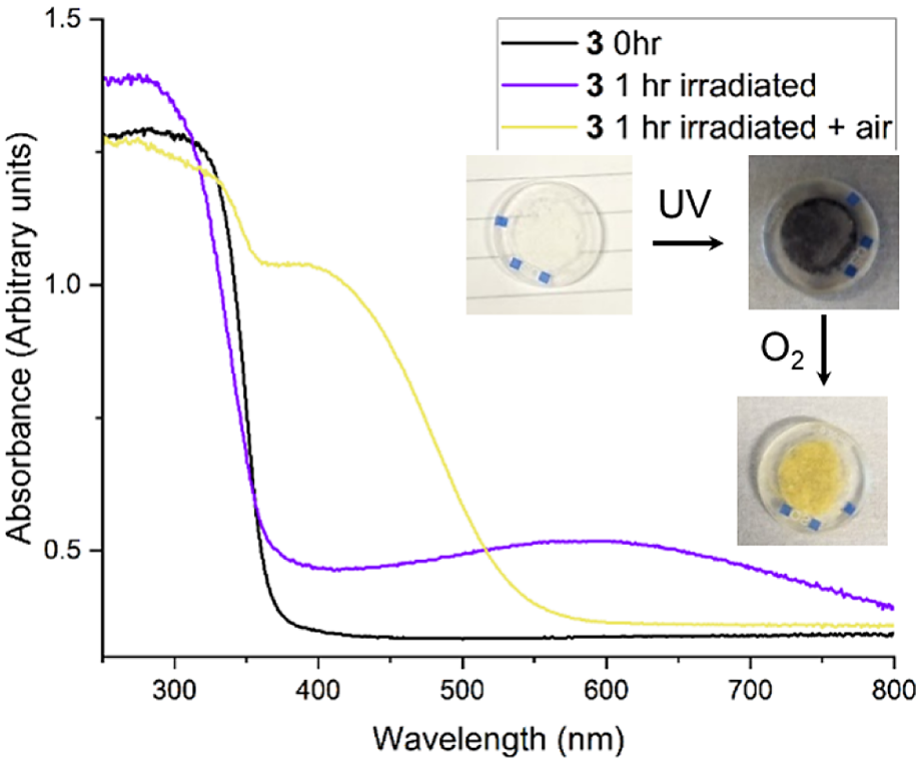
Diffuse reflectance UV/vis spectra and images of a powder of **3** before (white) and after UV irradiation (dark blue) and subsequently after exposure to air (yellow).

**1**, **2,** and **3** absorb UV light in solution or as powders in the solid state, with absorption onsets at ∼360 nm (Figures S1–S4).^19^ Powders of **1.py**, **2,** and **3** were subjected to 302 nm UV irradiation (∼3 mW/cm^2^ intensity). All these compounds show a color change from white to blue (Figures 2, S5–S6, N.B. powdered **1.py** gives a purple color, see Supporting Note 1). The resulting colored powders are very sensitive to rapid reoxidation under trace air to give yellow all-Ti^4+^ compounds (Figure 2);^18,19^ therefore, strictly inert conditions are necessary. To approximately quantify this photoprocess, the irradiated powders were subsequently dissolved in deuterated solvents and the integral of the acetone signal (the photooxidised product) was compared to the integrals of remaining starting cluster (Figures S7–S9). Despite significant color change, the results demonstrated a modest photochemical conversion after 6 h under the UV lamp (**1.py**, ∼6%; **2**, ∼9%; **3**, ∼3%). Single crystals of **1.py** and **3** were also found to undergo a clear color change to blue without loss of crystallinity when exposed to 302 nm. Diffraction data from these blue crystals returned structures consistent with the starting materials, in keeping with a small quantity of photoreacted clusters in the average structure. Therefore, to study this SC-SC transformation, a brighter UV source was required. The PORTO laser at the Diamond Light Source is a femtosecond pulsed laser system, comprising a PHAROS Laser 20 W ytterbium system from Light Conversion with an Orpheus HP optical parametric amplifier and second and fourth harmonics options. This provides a high-intensity variable wavelength laser light source, which can be overlapped with an X-ray spot, ideal for the in situ photoactivation of small single crystals. Furthermore, by mounting the crystals on the diffractometer (experimental hutch 2, beamline i19), the crystals can be protected from reoxidation by a cryostream of cold nitrogen gas (100 K), and laser irradiation and X-ray diffraction can be conducted without further handling of the crystal. Once mounted, the crystal was rotated during laser irradiation within the laser spot, and X-ray diffraction data sets suitable for structural solution were collected between various intervals of irradiation. Crystals of **1.py** (x2 crystals tested) and **3** undergo rapid color change from colorless to blue under 305 nm laser irradiation and retain crystallinity throughout the process. Diffraction quality slowly drops during the experiment, most likely due to strain-related microcracking of the crystal, which is commonly observed in SC-SC processes, and also possibly due to effects of X-ray beam damage (see Supporting Note 2).^61^ Despite this, the data quality is high enough for full structural solutions and refinement. The R factor of the refined solution increases slightly during this process (**1.py***R*_*1*_: before, 4.02%; after 900 s, 4.77%; **3***R*_*1*_: before, 3.93%; after 600 s, 5.31%). In the SC-SC products, bond length and angle metrics are most accurate near the core of the structure, while the carbon atom positions on the periphery show increasing degrees of disorder, which is typical in structures of metal-oxo clusters. Bearing this in mind, the Ti–O bonds are considered with the greatest importance in our analysis. While the data were collected over 40 min total irradiation (Figures S10–17), the optimal data sets representing the best balance of reaction completion versus diffraction quality were collected after 15 or 10 min (of **1.py** and **3** respectively), and these data sets are used for analysis of bond length data.

**Figure 3:**
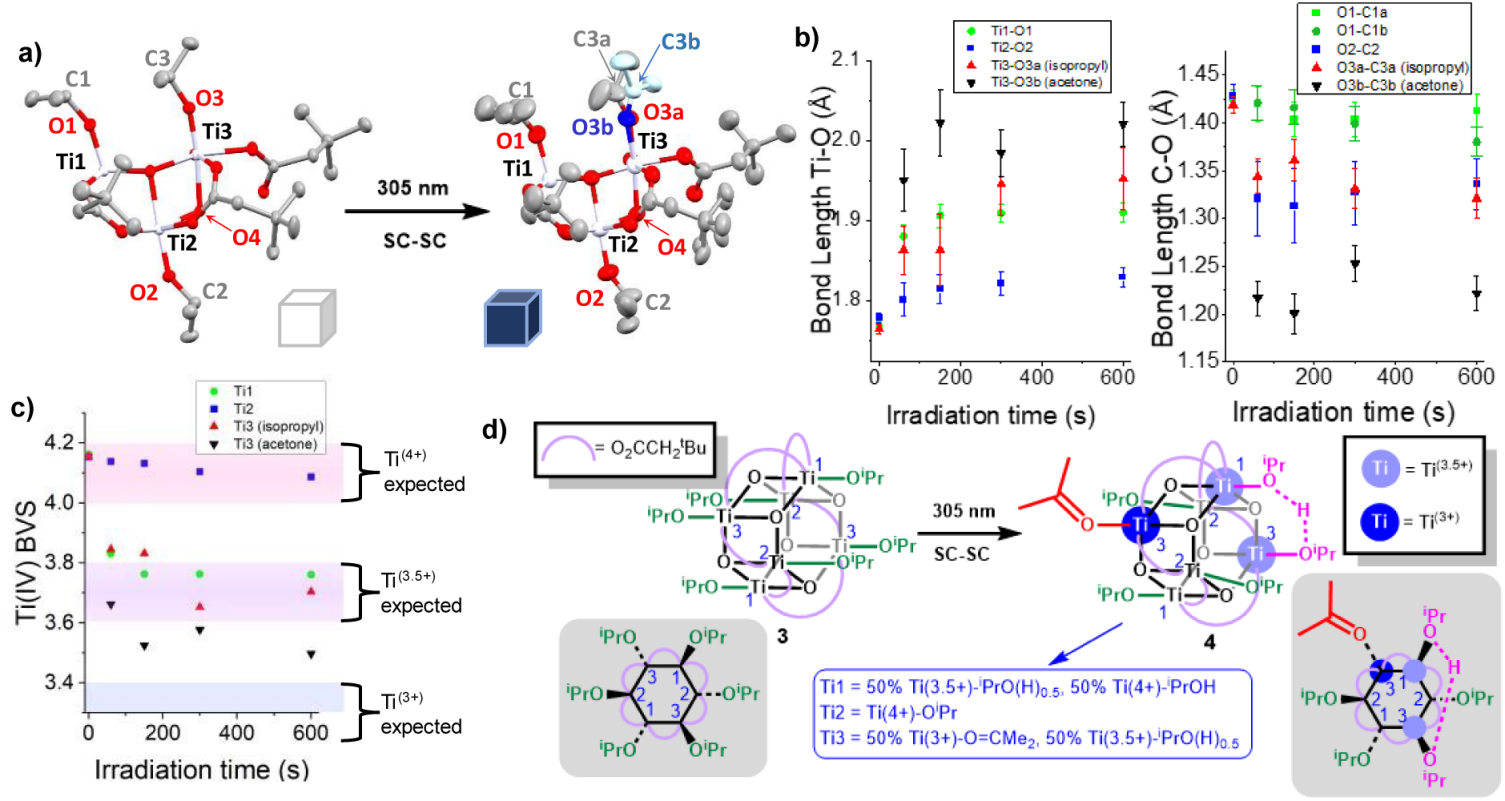
a) Solid-state structure of **3** before (left) and after 600 s of irradiation with a 305 nm laser (right). Displacement ellipsoids drawn at 50% probability. Titanium = lilac, oxygen = red, carbon = gray, disordered acetone fragment drawn in blue (oxygen = dark blue, carbon = light blue) only one asymmetric unit of the molecule is drawn, H atoms removed for clarity. Disordered O^i^Pr and ^t^Bu components were also observed (and shown) in the irradiated structure. b) Graphs showing structural parameters calculated from crystal structure models of **3** for each Ti environment. The Ti3 site is bifurcated into isopropyl (sp^3^ carbon) and acetone (sp^2^ carbon) fragments once irradiation is initiated. Error bars drawn at ±3σ. Left panel: Ti–O^i^Pr bond lengths. Right panel: O–C bond lengths in isopropoxide/acetone ligands. c) Bond valence sum calculation using expected geometry for Ti(IV) indicating a selective drop in oxidation state with three distinct regions assigned as Ti^4+^ (red shaded), Ti^3.5+^ (purple shaded), and Ti^3+^ (blue shaded) based on reported bond valence sum values from previously reported well-resolved related structures.^18,19^ d) SC-SC transformation of **3** → **4** and the predicted structure of **4**.

In the crystal structure of **3,** the Ti_6_ cluster sits on a center of inversion with half the molecule in the asymmetric unit; therefore, there are three crystallographically distinct Ti–O^i^Pr sites (Figure 3a). There is no positional disorder in the initial structure. Upon laser irradiation, significant changes are observed at the Ti3–O^i^Pr site, with more modest changes at Ti1–O^i^Pr, and a ^t^Bu group on the periphery, with these groups all requiring modeling over two sites in the final structure. The disorder components of the O^i^Pr units were not restrained to each other. The isopropoxide unit at the Ti3–O^i^Pr site becomes disordered, with the new disorder component exhibiting a short C–O bond length and a close to planar configuration (O–C–C–C torsion angle = 175(2)°), consistent with the formation of an acetone (C3b–O3b bond length ∼1.222(6) Å, compared to 1.21 Å for free acetone, or ∼1.23 Å for reported examples of acetone coordinated to Ti).^62−64^ The acetone fragment has a refined occupancy of ∼59%, broadly in keeping with one acetone per cluster at this site, noting that a value of 50% is expected at reaction completion due to the crystal symmetry. This implies that the photoreaction of **3** is essentially complete within 10 min (Figure 3a, b).^65^ This acetone ligand forms a long 2.020(9) Å bond to Ti3 as expected for a weakly coordinating neutral ligand (well-resolved literature examples range from 2.01 to 2.15 Å).^62−64^ The formation of acetone is expected to coincide with the protonation of another alkoxide on the cluster to give one neutral ^i^PrOH ligand, which would exhibit a longer O–Ti bond than a negatively charged isopropoxide. Previous studies have shown that the position of this alcohol proton can be disordered across the structure, and thus, averaged deviations in some of other O–Ti bond lengths may be expected.^19^ The alkoxide-like disorder component that remains at Ti3 (41% occupancy) exhibits an elongated Ti–O^i^Pr distance, compared to that in the starting structure, suggesting that the presence of a proton affects this position (Ti3–O3 distance, 1.764(2) Å → Ti3–O3a distance, 1.952(13) Å). The Ti3–O3a elongation is consistent with the bond lengths found in [Ti_6_O_6_(O^i^Pr)_4_(^i^PrOH)_4_(O_2_C^t^Bu)_6_] which has Ti–O distances of 1.955(3) and 1.940(3) Å for Ti–^i^PrO(H)_0.5_ groups associated with “half” a proton.^19^ The Ti3–O4 distance *trans* to the Ti-acetone fragment (O4 = μ_3_-O from the Ti-oxo core) has also decreased in the irradiated structure (Ti3–O4; 2.142(2) → 2.072(4) Å) supporting the coordination of a neutral *trans* ligand at this Ti site (Figure S11). The Ti1 site in irradiated **3** also shows a similar elongation of the Ti–OR distance (Ti1–O1 distance, 1.768(2) → 1.910(4) Å), which suggests that this alkoxide group also becomes influenced by the presence of the proton. This Ti1 site may in fact be a disordered mixture of O^i^Pr and ^i^PrO(H)_0.5_ ligands, if the proton is retained on one face of the cluster (see Figure 3d), consistent with the Ti–OR distance measured. In contrast, the “Ti2” site shows smaller bond length changes during the SC-SC transition, suggesting that this site is best described by retention of a Ti–O^i^Pr group (Figure 3b). It is noteworthy that the O–C bond lengths in all the O^i^Pr groups appear shortened after irradiation (Figure 3b), which indicates some further degree of disorder or imperfect modeling; however, all these O–C bond lengths remain larger than 1.34 Å, significantly different from the acetone fragment. Bond valence sum calculations reveal a significant oxidation state change at Ti3 and Ti1 (Figure 3c, Ti3 (acetone coordinated) calculated valence, 4.2 → 3.5; Ti3 (^i^PrO(H)_0.5_ coordinated), 4.2 → 3.7; Ti1, 4.2 → 3.8, using Ti(4+) parameters, see Figure S13 for analysis using Ti(3+) parameters). In contrast, a much smaller change is observed at Ti2 suggesting retention of Ti(4+) character (Ti2, 4.2 → 4.1). Collecting this evidence together suggests that the Ti3 site becomes 50% occupied by a Ti(3+) site coordinated to acetone and 50% occupied by a Ti(3.5+) site coordinated to ^i^PrO(H)_0.5_; the Ti1 site becomes 50% occupied by a Ti(3.5+) site coordinated to ^i^PrO(H)_0.5_ and the remaining sites at Ti2 and 50% of Ti1 retain Ti(4+) character coordinated to a O^i^Pr group (Figure 3d). This arrangement shows similarities with the structure of [Ti_6_O_6_(O^i^Pr)_4_(^i^PrOH)_4_(O_2_C^t^Bu)_6_], previously produced via the photoreduction of **2** in solution (after displacement of acetone).^19^ In summary, **3** undergoes an irreversible SC-SC transformation under UV light to form a photoreduced compound, **4**, best described as [Ti_6_O_6_(O^i^Pr)_4_(^i^PrOH)(OCMe_2_)(O_2_C^t^Bu)_6_], where the alcoholic proton may be delocalized or disordered over at least two sites. The structure suggests that the photoredox reaction results in protonation of the opposite side of the cluster to where the acetone is generated, which may occur via proton transfer steps. It is noteworthy that despite the chemical equivalence of all the Ti sites in **3** when in solution, the topochemical influence of the crystal lattice causes selective formation of acetone at only one of the three crystallographically distinct positions.^41,42^ In the crystal structure, the orientation of the isopropoxide groups vary and the α-proton on the Ti3–O^i^Pr fragment has the shortest distance to an O atom on a neighboring isopropoxide group (C3–H···O1 distance ∼2.7 Å), this may facilitate H atom transfer necessary for the formation of acetone and isopropanol following photoexcitation (Figure S18). Further analysis of the molecular Hirshfeld surfaces reveals differing contacts around the three crystallographically independent Ti–O^i^Pr sites in **3** (Figure S19).

**Figure 4:**
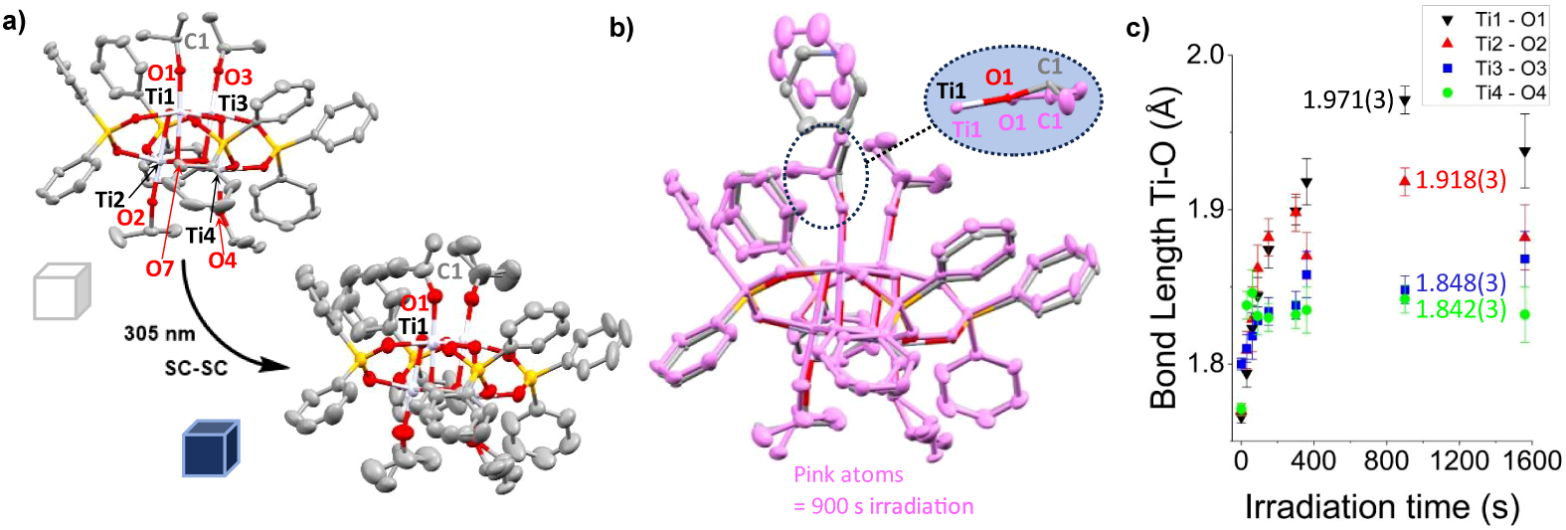
a) Crystal structures of **1.py** before (left) and after 900 s irradiation, with all disorder shown (right). Displacement ellipsoids drawn at 50% probability, and H atoms removed for clarity. Titanium = lilac, carbon = gray, oxygen = red, phosphorus = orange. b) Overlay of crystal structures of **1.py** before and after 900 s of irradiation. All atoms in structure after 900 s colored pink, displacement ellipsoids drawn at 20% and H atoms omitted for clarity. Dashed circle highlights the isopropoxide to acetone ligand transformation, with an alternative view of this Ti1–O1–C1(H_*x*_)Me_2_ section in the shaded circle (before (x = 1) and after (x = 0) irradiation). c) Graph showing Ti–O^i^Pr bond lengths from crystal structure models of **1.py** for each Ti environment. Error bars drawn at ±3σ. Bond length data after 900 s are displayed as text.

**Figure 5:**
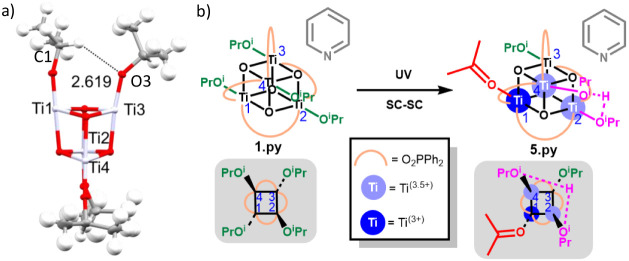
a) View of **1.py** showing close C–H···O distance (O_2_C^t^Bu ligands and pyridine are omitted for clarity). b) SC-SC transformation of **1.py** → **5.py** and suggested structure of the major disorder component of **5.py** (other disorder components expected to maintain structural arrangement but acetone located at a different Ti position).

Crystals of **1.py** also undergo a similar SC-SC process. The structure of **1.py** has all four Ti positions sitting in crystallographically independent sites and has no positional disorder (Figure 4a). Throughout the SC-SC process, some displacement and disorder of the [Ph_2_PO_2_]^–^ ligands is observed, including a clear rotation of one phenyl group by 40.6° (C52–P44–C46–C51 torsion angle 27.4(2)° → 70.0(4)°). All the titanium isopropoxide fragments change in their structural parameters (bond lengths and angles); however, each of the four crystallographically distinct isopropyl sites displays differing changes upon irradiation. After irradiation, three isopropoxide sites (at Ti2, Ti3, and Ti4) are modeled over two disorder positions. However, it was not possible to further model each site as the expected disordered mixture of isopropoxide, isopropanol, and acetone; therefore, these three sites are modeled as averaged O^i^Pr fragments across two sites. In contrast, the O^i^Pr group at Ti1 changes to a clearly resolved (close-to) planar geometry (O–C–C–C torsion angle = 170(1)°) associated with acetone as the major component at this position (Figure 4b). The Ti1–O1 bond length increases, inferring a neutral ligand at this position (Ti1–O1 distance, 1.7657(13) → 1.971(3) Å), and the *trans* Ti1–O7 distance in the core of the cluster is associatively reduced (2.150(1) → 2.071(3) Å, Figure 4c, S15). The Ti2 site also shows elongation of the average Ti–O bond distance, inferring the presence of a mainly neutral ligand at this site, while Ti3 and Ti4 show smaller changes consistent with a mixture of ^–^O^i^Pr and ^i^PrOH ligands (Figure 4c). The C1–O1 bond length at Ti1 shows a major decrease and approaches the bond length expected for acetone (Ti1, 1.4211(18) → 1.275(6) Å) (Figure S12). Due to the multicomponent disorder at the other sites, further C–O bond length analysis was not possible. The change in bond lengths and the deformation of the O^i^Pr at Ti1 to a nearly planar fragment (which is well resolved, with smaller displacement ellipsoids than all other O^i^Pr groups in the final model, Figure 4b) suggest that the electron density at Ti1 is mostly from the presence of acetone at this site, while a smaller fraction of acetone may be located at the Ti2 site as a disordered mixture. Disordered O^i^Pr/^i^PrO(H)_0.5_ is expected at the other sites. In the starting structure of **1.py** the O^i^Pr fragment at Ti1 is well positioned for H atom transfer to a neighboring alkoxide (C1–H···O3 distance ∼2.6 Å, Figure 5a), while the O^i^Pr fragment at Ti2 shows a slightly longer distance (∼3.5 Å) but with a similar orientation (Figure S20), therefore, this intramolecular C–H···O proximity appears to correlate with likelihood of acetone formation.

The pyridine solvent molecule is retained approximately at its original position in the crystal lattice (with a slight positional shift and rotation, Figure 4b) although it becomes increasingly disordered. Freely refining the pyridine occupancy during the SC-SC process resulted in a modest drop in occupancy to ∼90% although this could be an artifact of the increasing disorder. In the single crystal environment, under these low-temperature (100 K) irradiation conditions, there is no evidence that the pyridine is able to move within the crystal lattice to displace the acetone ligand and coordinate to the Ti cluster, which is observed during analogous studies in solution (see Supporting Note 1 for comparative discussion of powder samples at room temperature).^18,19^

Bond valence sum calculations using these models imply a decrease in oxidation state across **1** although to varying degrees (Ti1, 4.2 → 3.6; Ti2, 4.2 → 3.7; Ti3, 4.1 → 3.9; Ti4, 4.2 → 4.0, Figure S17). While the data from irradiated **1.py** give less confidence for determining oxidation state and proton location in comparison to the structure of **4**, we tentatively describe the oxidation state distribution in the same way, i.e., one site as a Ti(3+), and two sites with Ti(3.5+) character, but with some disorder of these positions (Figure 5b). Therefore, the product of the photoirradiation of **1.py** is best described as [Ti_4_O_4_(O^i^Pr)_2_(^i^PrOH)(OCMe_2_)(O_2_PPh_2_)_4_]·C_5_H_5_N, **5.py**, where the acetone is found predominantly at one Ti site but with some disorder.

## Spectroscopic Studies

Irradiated powders of **1.py** and **3** were also investigated by spectroscopic methods. These methods necessitated the use of bulk samples, and so laser irradiation was no longer possible; therefore, due to slower photoreactivity, only small quantities (∼6%) of the photoredox products **5.py** and **4** were produced as a mixture with the respective starting materials. EPR spectroscopy displayed broad signals and confirmed the presence of new paramagnetic species upon irradiation in both systems (**5.py**, 292 K; g = 1.948; **4**, 292 K; g = 1.951), consistent with a system containing titanium(3+) (Figures S21–S25).^18,66^ A low temperature spectrum (145 K) of **4** revealed a clear signal at g ∼4 (Figure S25) alongside the broad signal at g ∼1.95, which is indicative of a triplet state (*S* = 1), and consistent with the expected two electron photoreduction of **3** into **4**. These signals at lower field have also been previously observed on irradiated solutions of **1**.^18^ After exposure to air **4** and **5.py** both form yellow compounds (Figures S5–S6) which exhibit EPR spectra at g_⊥_ = 2.007 and g_∥_ = 2.02 and with no further Ti-based signals (Figures S26–29) suggesting the formation of superoxide salts upon oxidation of the powder (e.g., **5.py** transforming into [Ti_4_O_4_(O^i^Pr)_2_(^i^PrOH)(OCMe_2_)(O_2_PPh_2_)_4_][O_2_]_2_·C_5_H_5_N). This is consistent with the previously reported aerobic oxidation of solid [Ti_4_O_4_(O_2_PPh_2_)_6_] into [Ti_4_O_4_(O_2_PPh_2_)_6_][O_2_]_2_.^18^

Solid-state diffuse-reflectance UV spectroscopy was conducted on powders of **1.py** and **3** before and after irradiation. The measured absorption onsets of both **1.py** and **3** are similar to those from solution studies (**1** solution, 361 nm; **1.py** solid, 365 nm; **3** solution, 359 nm; **3** solid, 367 nm). The solid spectra of the irradiated powders show broad absorptions across the visible spectrum indicative of d-d transitions or intervalence charge transfer excitations (Figures 2, S5–S6). The formation of acetone in the solid-state photoproducts was confirmed by the NMR spectra of photoactivated powders after they were dissolved (Figures S7–9). Upon oxidation, **4** and **5.py** convert to yellow compounds, with absorptions into the visible region consistent with reported titanium superoxide (or peroxide) compounds.^18,19^

**Figure 6:**
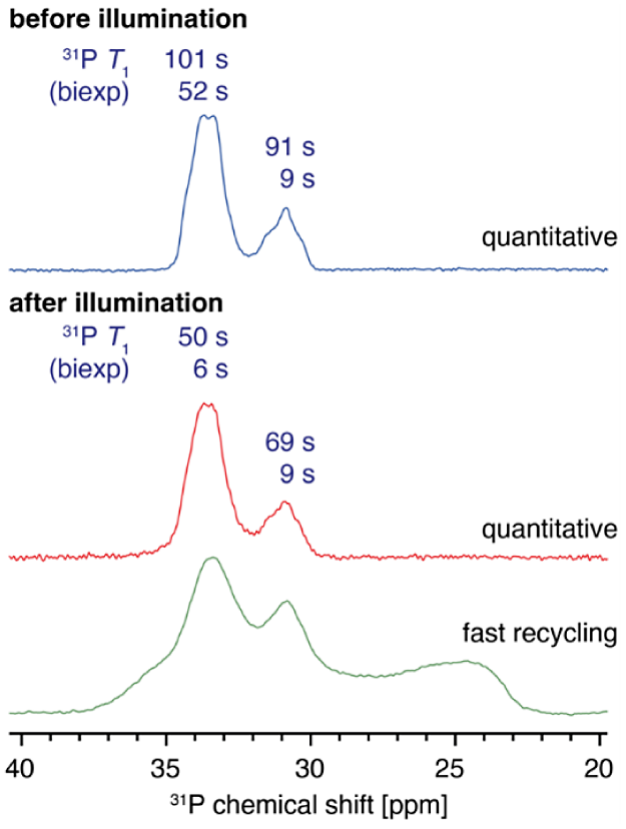
^31^P echo-detected MAS NMR spectra of **1.py** before and after illumination recorded at 23 T, 30 kHz MAS and 298 K. The two components of the biexponential ^31^P *T*_1_ fit are given above each signal.

Solid-state magic angle scattering (MAS) NMR was used to probe how the local structure of **1.py** changes upon irradiation. The formation of paramagnetic Ti(3+) is expected to lead to changes in the line shape (through hyperfine shifts) and relaxation (through paramagnetic relaxation enhancement, PRE) owing to the interaction of the nuclear spins with the unpaired electrons. While the quantitative ^1^H MAS NMR spectra of the material before and after illumination are identical, the ^1^H longitudinal relaxation time, *T*_1_, is substantially shortened in the irradiated material (^1^H *T*_1_ = 1.4 and 0.3 s, biexponential) compared to the pristine material (^1^H *T*_1_ = 2.7 s, monoexponential), which clearly shows the presence of PRE (Figure S30). In protonated solids, the strong ^1^H–^1^H dipolar interactions result in fast spin-diffusion (SD), which homogenizes the magnetization within the crystallites leading to an average monoexponential *T*_1_.^67,68^ The biexponential nature of ^1^H *T*_1_ in the illuminated sample indicates that *SD* does not operate uniformly across the sample, thereby suggesting a strongly nonuniform distribution of paramagnetic centers within the crystallites. The shorter and longer ^1^H *T*_1_’s correspond, respectively, to regions with higher and lower concentrations of the paramagnetic Ti(3+) species. This finding agrees with the nonuniform distribution of the photoreduced species observed visually, whereby the dark blue color is confined to the surfaces of a powdered sample (which becomes paler on grinding), resulting from an incomplete conversion (6% for **1.py**, as quantified by liquid-state NMR). The *T*_1_ shortening caused by Ti(3+) PRE after illumination is also evident for ^31^P in **1.py** (Figure 6). In this case, the ^31^P *T*_1_s for the pristine material are best fitted with a biexponential function because each peak contains multiple local environments characterized by different relaxation rates. Since ^31^P–^31^P *SD* is much less efficient compared to ^1^H–^1^H SD, it does not lead to a single *T*_1_ across the material. The ^31^P spectrum of pristine **1.py** shows two signals, with an area ratio of 3:1, corresponding to the four unique ^31^P sites in the asymmetric unit cell. Owing to the similarity of the four local environments and the large unit cell size, we were not able to assign the signals using chemical shift calculation at this time. While owing to the small degree of conversion the quantitative ^31^P spectra before and after illumination are nearly identical, the signal of the local environments that are in the close proximity of the paramagnetic Ti(3+) centers, and therefore affected by PRE to the highest extent, can be enhanced by recording a spectrum using a short recycling delay. Consequently, the slowly relaxing local environments unaffected by PRE do not have enough time to recover and the spectrum is dominated by the fast-relaxing species. A spectrum recorded in this way evidences the presence of new local environments (for **5.py**) which are substantially shifted relative to the pristine ones. This shift is caused by the through-space interaction between the ^31^P nuclei and the unpaired electrons on the Ti(3+) center (pseudocontact shift, PCS) at 3.2 Å, and unambiguously evidences the formation of Ti(3+) species. The maximum magnitude of the PCS, up to about 10 ppm, is consistent with that calculated for typical Ti(3+) species.^69^^13^C spectra were also recorded which show the presence of the aromatic O_2_PPh_2_ ligand and the O^i^Pr groups (Figure S30).

## Conclusions

The crystalline Ti-oxo clusters **1.py** and **3** undergo a single-crystal to single-crystal transformation under UV irradiation, which is studied directly by single-crystal diffraction techniques. Photochemical activation initiates a two-electron redox process resulting in the oxidation of isopropoxide to acetone and two-electron reduction of the titanium-oxo core, which is observed by EPR spectroscopy. These clusters are able to undergo a multielectron process, due to the multiple titanium sites and the flexibility of the ligand environment. Within the confines of the solid state, the coproduced acetone photoproduct remains coordinated to the reduced cluster, providing precise reaction information beyond what is possible by solution studies, in which ligand exchange occurs readily. These solid-phase results further support the proposed mechanism of a two-electron intramolecular photoredox reaction of Ti-oxo clusters, without evidence of free organo-radical formation. These findings promote the prospect of utilizing multimetallic complexes of earth-abundant early transition metals in multielectron (photo)catalytic processes. These results also provide precise reactivity information relevant to the photoredox reactivity of TiO_2_ nanoparticles and Ti-based MOFs.^11−14^

Remarkably, compound **3** shows selective reactivity at one crystallographically independent Ti–O^i^Pr site, despite the fact that all of the sites are chemically identical. Site selective photoreactivity is also observed in **1.py**. The crystalline environments desymmetrise these molecules and the structures suggest that a short intramolecular distance between the α-proton and O atom of adjacent isopropoxide groups may be important to facilitate internal H atom transfer as part of the redox transformation to form acetone and isopropanol.^18^ Further influences could arise from the light-crystal orientation, including differential absorption profiles of different crystal faces, and these aspects may be of future interest. These results demonstrate the possibility for selective photoreactivity in the crystal phase, perhaps controllable by crystal engineering. Modification of cosolvents or selection of differing polymorphs may promote certain site selective reactivity by creating differing steric and electronic local environments in the crystal.

## Data Availability

The raw data that support the findings of this study are available from https://wrap.warwick.ac.uk/186369.

## Supporting Information Available

Experimental details, preparation and characterization of compounds, crystallographic refinement information, crystallographic data and figures, and spectroscopic figures (PDF)
X-ray crystallography data for the irradiation process (CIF)

## Supplementary Material

ja4c04068_si_001.pdf

ja4c04068_si_002.cif
