# Supplementary material for: Photoinitiated Single-Crystal to Single-Crystal Redox Transformations of Titanium-Oxo Clusters
Source: J Am Chem Soc. 2024 Jun 12;146(25):17325–33. doi: 10.1021/jacs.4c04068 (PMC11212046; doi:10.1021/jacs.4c04068)
Supplement: Supplementary file 1 — ja4c04068_si_001.pdf [file ja4c04068_si_001.pdf]

Photoinitiated Single-Crystal to Single-Crystal Redox Transformations of Titanium-Oxo Clusters.

Stephen E. Brown,[1] Mark R. Warren,[2] Dominik J. Kubicki,[3] Ann Fitzpatrick,[4] Sebastian D. Pike*[1]

1. Department of Chemistry, University of Warwick
2. Diamond Light Source, Harwell Science & Innovation Campus
3. School of Chemistry, University of Birmingham
4. RAL Space, Harwell Science & Innovation Campus

Contents

Experimental details 1

Preparation and characterisation of compounds 3

Notes on SCXRD crystal refinement methodology 3

Supporting characterisation figures & tables 5

Supporting Note 1. Exploration of co-crystallised solvent molecules 30

Supporting Note 2. Evaluation of X-ray beam damage 32

Crystallography data tables 33

References 37

Experimental details

All manipulations were carried out under a nitrogen atmosphere using Schlenk line techniques or a glovebox unless otherwise stated. Diphenylphosphinic acid and $Ti(O^iPr)_4$ were used directly from suppliers. $Ti(O^iPr)_4$ is a liquid and was weighed (negative mass of donor flask) and transferred by syringe within a glovebox. 'Extra-dry' acetone and isopropanol were purchased from Acros Organics, 'anhydrous' toluene and THF was purchased from Sigma Aldrich. 3,3-dimethylbutyric acid ($^tBuCH_2COOH$) and DMSO were dried over activated 4 Å molecular sieves; d_8-toluene, d_6-benzene, pyridine and acetonitrile were dried by stirring over CaH_2 and vacuum distilled using grease-free trap-to-trap apparatus, before storing over molecular sieves under nitrogen. All solvents were degassed by three freeze-pump-thaw cycles and stored over 4 Å molecular sieves (3 Å molecular sieves for acetone, isopropanol and acetonitrile).

Moisture analysis of prepared solvents was conducted by Karl Fischer spectrometric analysis:

Solvent	Water content (ppm)
MeCN	8.4
^{i}PrOH	15
Pyridine	8.8
THF	9.9
Et_2O	31
Toluene	5.6

Solution NMR spectra were recorded on Bruker Avance III HD 400 MHz or 300 MHz instruments and all chemical shifts reported in parts per million (ppm).

Solution UV/visible spectroscopy was recorded using a Implen NanoPhotometer C40, using a bespoke Young's tap cuvette for air free analysis.

UV Irradiation of powder samples was undertaken within sealed glass tubes (or quartz tubes for EPR spectroscopy) under inert atmosphere. The tube was shaken periodically to ensure powder was irradiated from different angles. The powders become coloured upon irradiation, but this colour appears to fade upon grinding, indicating that colouration occurs mainly on the surface of particles.

Solid State UV-Vis-NIR spectroscopy was done on a Shimadzu 2600i in diffuse reflectance mode with an integrating sphere attachment, the absorbance was calculated using the Kubelka-Munk function and was subtracted from a baseline of $BaSO_4$. These measurements were carried out air free by loading the sample into 50 μm deep quartz disk cuvettes inside a glovebox which were sealed with vacuum grease and clamped together. This setup was useful for determining the qualitative absorption spectrum of the sample, however, due to some light leakage from the air-free setup, analysis of the absolute absorbance relative to $BaSO_4$ (y-axis) is less accurate.

EPR spectroscopy was carried out at X-band (~9.5 GHz) on a Bruker EMX spectrometer fitted with a variable temperature cryostat on powdered samples at room and cryogenic temperatures. Spectrometer settings: microwave power, 50 mW; microwave attenuator, 6 dB; time constant, 20.48 ms; conversion time, 20.48 ms; modulation frequency, 100 kHz, and amplitude, 1G. Samples were irradiated inside the young's tap quartz EPR tubes. EPR data was modelled using the EasySpin toolbox for MATLAB.

^{1}H (1000.40 MHz), ^{31}P (404.97 MHz) and ^{13}C (251.55 MHz) solid-state MAS NMR spectra were recorded at room temperature on a Bruker Avance Neo 23.0 T spectrometer equipped with 1.9 mm MAS probe and referenced to the ^{13}C signal solid adamantane (δ_{CH} = 38.48 ppm) as per the IUPAC recommendation.[1] The following recycle delays and numbers of scans were used: ^{31}P echo: 600 s (quantitative, **1.py**, starting product, 32 scans), 150 s (quantitative, **1.py**, photoreduced, 48 scans), 0.5 s (fast recycling, **1.py**, photoreduced, 10240 scans); ^{1}H echo: 13 s (quantitative, **1.py**, starting product, 4 scans), 5 s (quantitative, **1.py**, photoreduced, 4 scans); ^{13}C echo: 1 s (**1.py**, starting product, 1873 scans), 1 s (**1.py**, photoreduced, 5670 scans); The RF strengths were 83 kHz (^{1}H), 45 kHz (^{31}P), 45 kHz (^{13}C). 80 kHz of ^{1}H decoupling was used for all ^{13}C and ^{31}P spectra. The rotors were packed in a nitrogen glovebox.

X-ray diffraction of photoirradiated crystals experiments: Measurements were carried-out at Diamond Light Source on beamline I19-EH2 using a wavelength of 0.48590 Å and a four-circle Newport diffractometer equipped with a Dectris Eiger CdTe X 4M detector. Crystals were taken from a flask under a flow of nitrogen and mounted onto MiTeGen UV-Vis™ loops under fomblin-Y oil swiftly in air before being placed on the goniometer under a 100K nitrogen stream. Crystals were photoirradiated *in situ* using the PORTO variable wavelength pulse laser (305 nm, 800 mW/cm^2). During irradiation the crystals were rotated. The laser and X-ray spot were overlapped on the sample position. X-ray diffraction measurements were carried out in between laser irradiation intervals.

The PORTO is a femtosecond pulsed laser system comprising of a PHAROS Laser 20 W Ytterbium system from Light Conversion with an Orpheus HP optical parametric amplifier and 2nd and 4th harmonics options. It has a 50 KHz repetition rate, 300 fs pulse and a power at sample position of 1.3 mW (measured by a Thorlabs power metre 5405C, 1906222, 5W). The Beam size (knife edge scans) is: Horizontal size: FWHM 280 um; Vertical: FWHM 520 um (measured using SM1PD1A Thorlabs).

Preparation and characterisation of compounds

1.tol: $[TiO(O^iPr)(OOPPh_2)]_4.(PhMe)$

1.tol was prepared following the published procedure.[2]

1.py: $[TiO(O^iPr)(OOPPh_2)]_4.(C_6H_5N)$

580 mg (0.399 mmol) of **1.tol** $[Ti_4O_4(O^iPr)_4(O_2PPh_2)_4(PhMe)]$ was dissolved in a minimal amount (10 mL) of hot pyridine and then cooled to −20 °C. The remaining solution was decanted leaving 391 mg (0.272 mmol, 68% yield) of colourless crystals of **1.py**.

3 $[Ti_6O_6(O^iPr)_6(O_2CCH_2{}^tBu)_6]$:

N.B. **3** has been previously reported using a solvothermal reaction route.[3]

2.04 g (17.6 mmol) of 3,3-dimethylbutyric acid was put into a nitrogen filled Schlenk flask and dissolved in 15 mL of toluene. This solution was added dropwise, over 10 minutes, to a Schlenk flask containing 5.00 g (17.6 mmol) of $Ti(O^iPr)_4$ in 10 mL of toluene and stirred for 30 mins. 322 µL (17.6 mmol) of H_2O was dissolved in 3 mL of dry acetone in a separate flask and this mixture added slowly to the reaction flask over 30 mins. The reaction solution was stirred at 60 °C for 18 hrs before the solution was reduced to approximately one third of its volume under vacuum and then stored at −20 °C to yield 2.12 g (1.49 mmol, 51 % yield) of white, highly crystalline product **3**.

^1H NMR spectroscopy (C_7D_8, 400 MHz): δ 5.14 (6H, sept (J_{HH} = 6 Hz), OC**H**$(CH_3)_2$); 2.20 (12H, s, $O_2CC\mathbf{H}_2$ $(CH_3)_3$; 1.57 (36H, d (J_{HH} = 6 Hz), $OCH(C\mathbf{H}_3)_2$); 1.13 (54H, s, $O_2CCH_2(C\mathbf{H}_3)_3$)

^1H NMR spectroscopy (C_6D_6, 400 MHz): δ 5.19 (6H, sept (J_{HH} = 6 Hz), OC**H**$(CH_3)_2$); 2.26 (12H, s, $O_2CC\mathbf{H}_2$ $(CH_3)_3$; 1.61 (36H, d (J_{HH} = 6 Hz), $OCH(CH_3)_2$); 1.16 (54H, s, O2CCH2$(C\mathbf{H}_3)_3$)

Notes on SCXRD crystal refinement methodology

For all data sets spotfinding and integration were done using the DIALS automated pipeline,[4] unirradiated crystal structures (time = 0 s) were solved using SUPERFLIP[5] and refined using CRYSTALS[6] implementation of SHELXS.[7] Later timepoints used the time = 0 s model as an initial solution and were refined in the same way.

During the photoirradiation experiment, a complete 360° omega scan was performed at each timepoint, which gave a good compromise between completeness, redundancy and collection time and beam dose for the samples with the P 2/m Laue class.

Refinement and disorder model of **1.py**.

Two separate crystal series were collected for **1.py** on two different crystals. As the diffraction data were similar from each crystal and show concordant changes on irradiation the time series includes data from both crystals. The time series on crystal one is t = 0 s, 60 s, 360 s, 1560 s, 6160 s, whereas for crystal two is t =30 s, t= 90 s, t = 150 s, t = 300 s and t= 900 s. Note that comparison of the initial data collections of each crystal, crystal 1 (no irradiation) and crystal 2 (t = 30 s), is helpful for differentiating photochemical conversion from any sequential X-ray induced beam damage (see supporting note 2).

In general, to allow for greater comparisons between structures after different irradiation times the same disorder model was used across all structures in the series except for the initial t = 0 s structures (which do not exhibit disorder). When the disorder model is forced on to the initial structure occupancy of disordered fragments tends towards zero (or negative) and the resulting thermal ellipsoids become non positive definite. This is symptomatic of the disorder model not being appropriate for the initial structure as the photogenerated disorder is not present at time = 0 s.

Because of the chemical similarity of each isopropoxide unit it is reasonable to propose that the resulting acetone and isopropanol (and remaining isopropoxide) could be disordered in each of the four sites. Notably, each site evolves differently under irradiation and so a disorder model was used that reflected this. Ti4 – O81 – C82 [Ti4 – O4 – C4 in main text] is modelled as one fragment with complete occupancy, no restraints or constraints on geometry are applied. All other titanium isopropyl fragments are split at their carbons and modelled as two parts sharing a common unsplit oxygen. It is necessary to apply geometry restraints to the resulting fragments to have a convergent structure and these were chosen with great care. Within each part of the OC_3 ligand fragment the geometry of each half of the fragment is restrained as similar using SAME, SIMU and DELU restraints, as a reflection plane is chemically sensible for any of acetone/isopropoxide/isopropanol. However, no geometry restraints are applied *between* each part, this means each part is not restrained to being similar to any other part and can have differing geometry. This was done to allow for the possibility of an acetone fragment and isopropoxide/isopropanol fragment being co-located in the average structure. Further disorder modelling was necessary for two phenyl rings which are disordered in the photoproduct structure. These are modelled as two split parts and restrained with equivalent geometry as there is no reason to expect chemical difference. Finally, restraints were placed on the co-crystallised pyridine solvent molecule which shows significant disorder as irradiation time increases (noting that it is well-resolved at t=0).

In the analysis of bond lengths within this paper the model without disorder is used for before irradiation (t=0) and all other datasets are treated with the disorder model.

Refinement and disorder model of **3.**

3 was modelled in a similar method to **1.py**. In the irradiated structures two isopropoxide/isopropanol/acetone fragments on the Ti1 and Ti3 positions were split, whilst the isopropoxide at Ti2 remains unsplit. Each component of the disordered isopropoxide/isopropanol/acetone was restrained to have two-fold symmetry within the molecule but with no restraints between each part. Additionally, one tert-butyl group was modelled over two sites with restraints including similarity between parts. The occupancy of the acetone component (at Ti1) grows to ~0.4 after 60 s irradiation, before rising to ~0.5 after 150 s and settling at ~0.6 at 300 s or longer. Due to the symmetry of the molecule, the acetone occupancy is expected to reach 0.5 on completion of reaction. Forcing the acetone fragment to an occupancy of 0.5 has little effect on the structure but resulted in less satisfactory atomic displacement parameters, and, therefore, free refinement of occupancy was used.

In the analysis of bond lengths within this paper the model without disorder is used for before irradiation (t=0) and all other datasets are treated with the disorder model.

Supporting Characterisation Figures & Tables

Table S1. Observations during photoirradiation of powdered samples

Compound	Time / hours	Colour of powder after irradiation	Photoconversion (%, by analysis of acetone peak in NMR spectrum in comparison to starting material after dissolving the irradiated sample)
**1.tol**	6	Pale grey	13
**1.THF**	1.5	White	Not recorded
**1.DMSO**	6	Pale grey	Negligible
**1.MeCN**	1.5	Pale grey	Not recorded
**1.HO^{i}Pr**	6	Pale Blue	3
**1.py**	6	Pale Purple	6
**1.py**	20	Purple	12
**2**	6	Blue	9
**3**	6	White	3

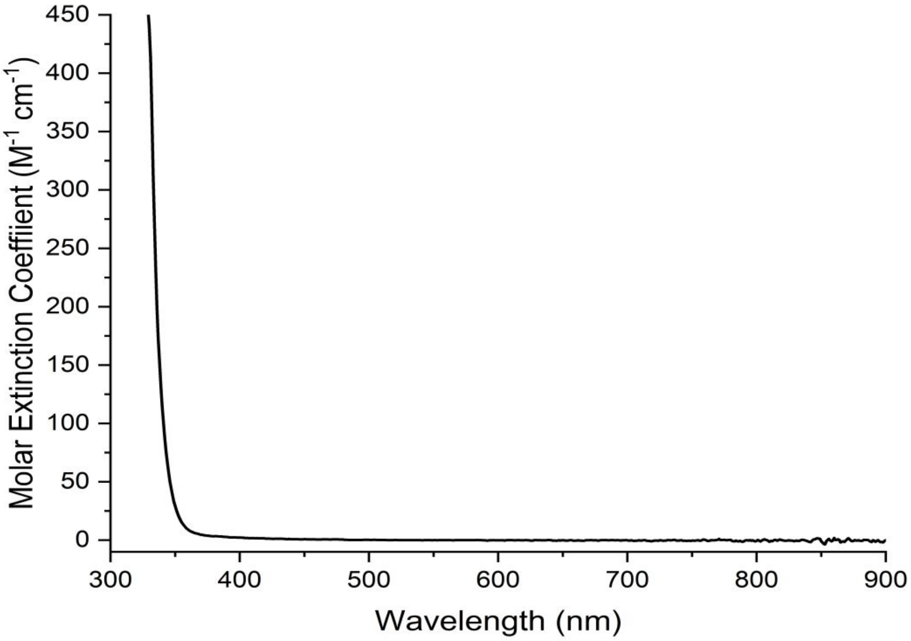

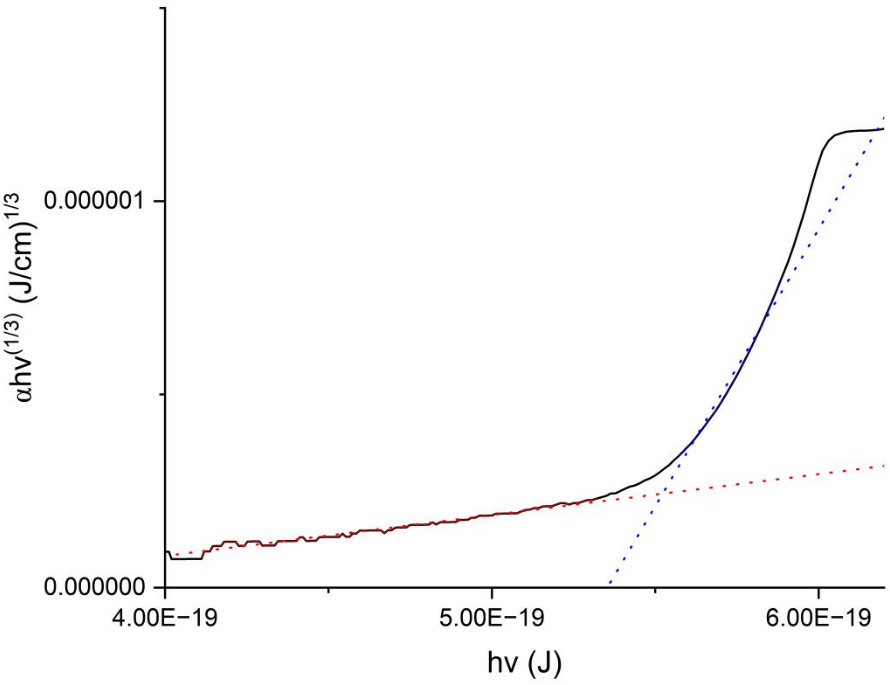

Figure S1. UV-Vis absorption plot of a 5.83 mM Solution of **3** in toluene ([Ti] concentration = 35 mM) (top) and Tauc plot analysis giving an absorption onset of 359 nm, 3.46 eV (bottom).

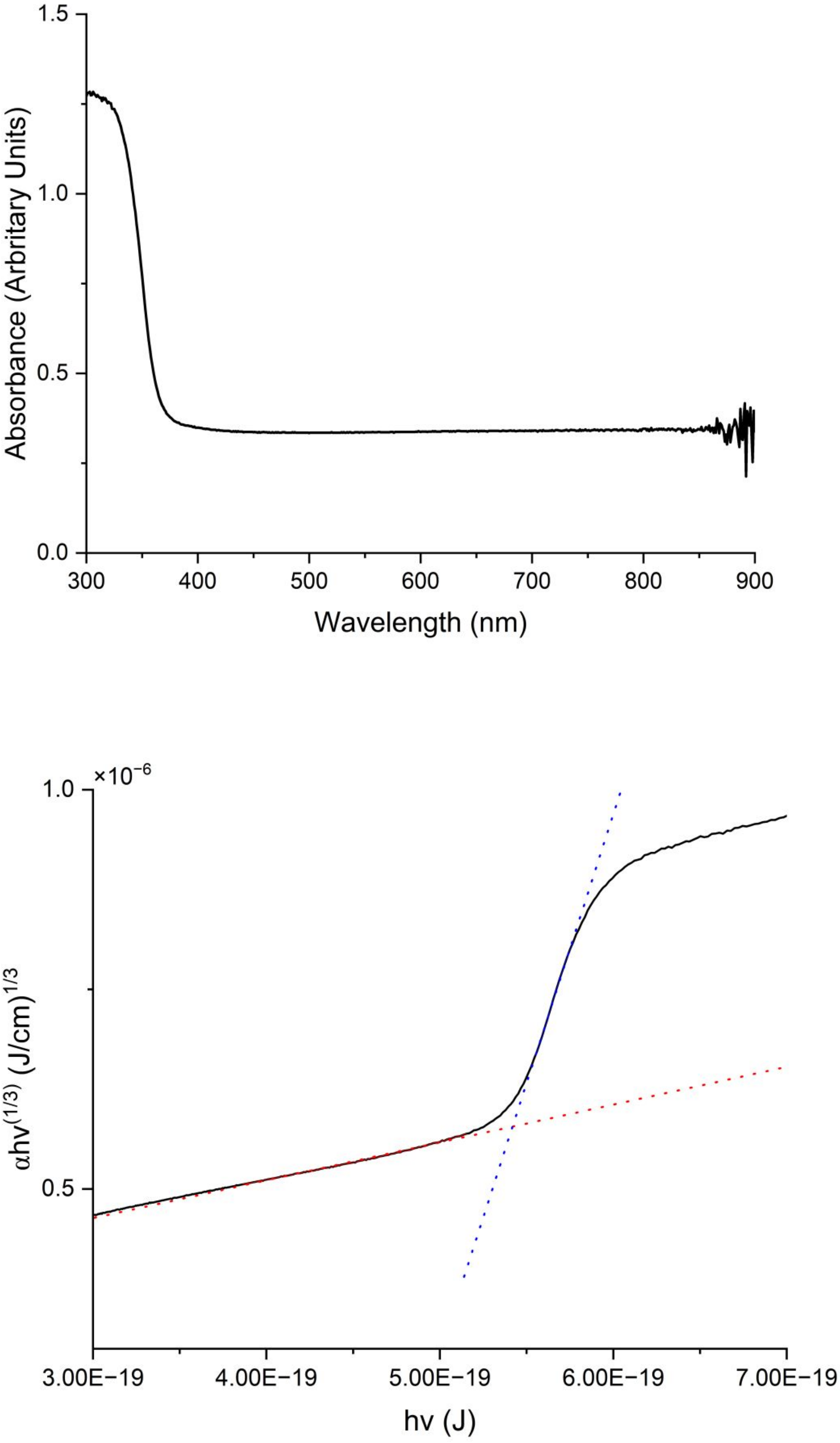

Figure S2. Diffuse reflectance UV-Vis absorption plot of **3** as a powder (top) and Tauc plot analysis giving an absorption onset of 367 nm, 3.38 eV (bottom). Note that the baseline is shifted upwards relative to the reference sample due to some light leakage associated with the use of an airtight sample holder in the diffuse reflectance experiment.

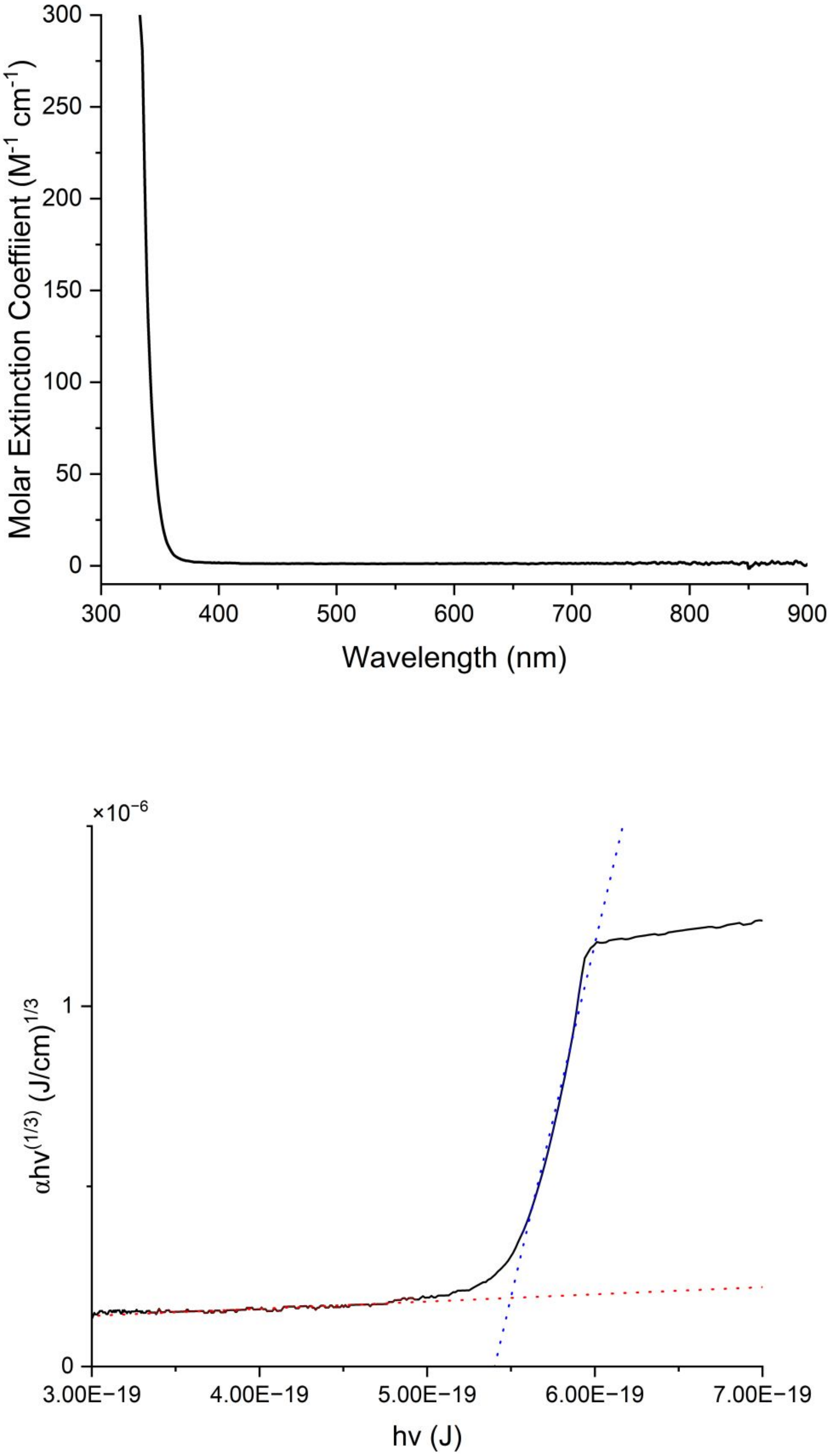

Figure S3. UV-Vis absorption plot of a 8.75 mM solution of **1** in toluene ([Ti] concentration = 35 mM) (top) and Tauc plot analysis giving an absorption onset of 362 nm, 3.44 eV (bottom).

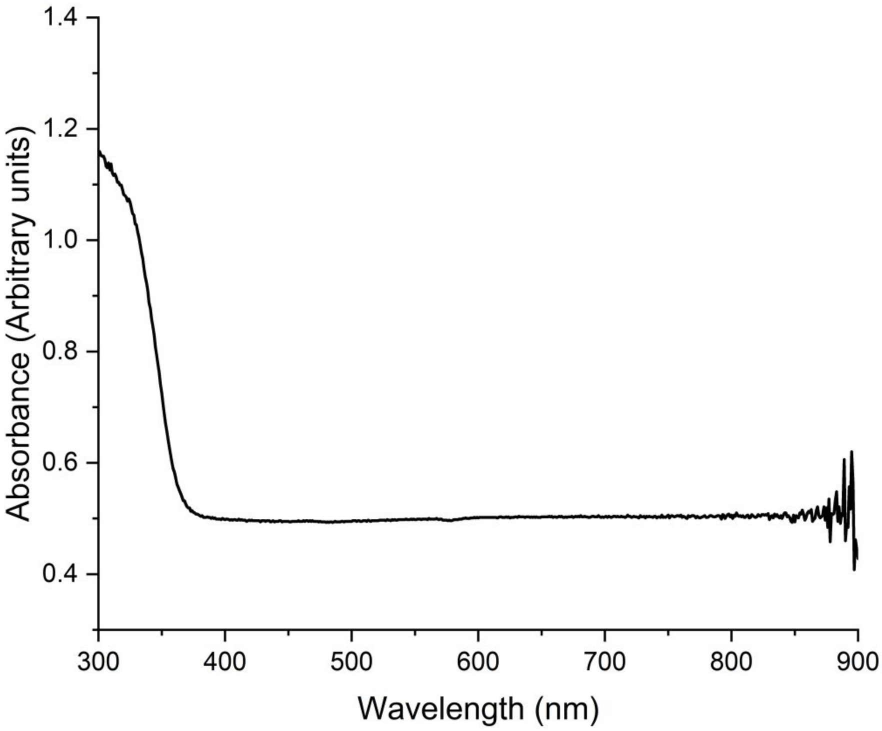

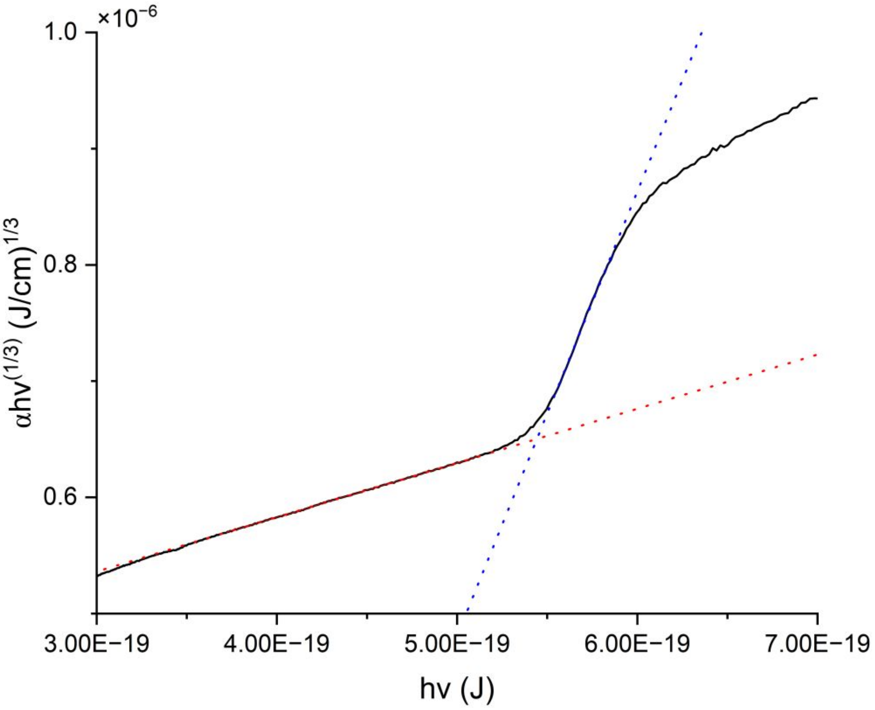

Figure S4. UV-Vis absorption plot of **1.py** as a powder (top) and Tauc plot analysis giving an absorption onset of 365 nm, 3.40 eV (bottom). Note that the baseline is shifted upwards relative to the reference sample due to some light leakage associated with the use of an airtight sample holder in the diffuse reflectance experiment.

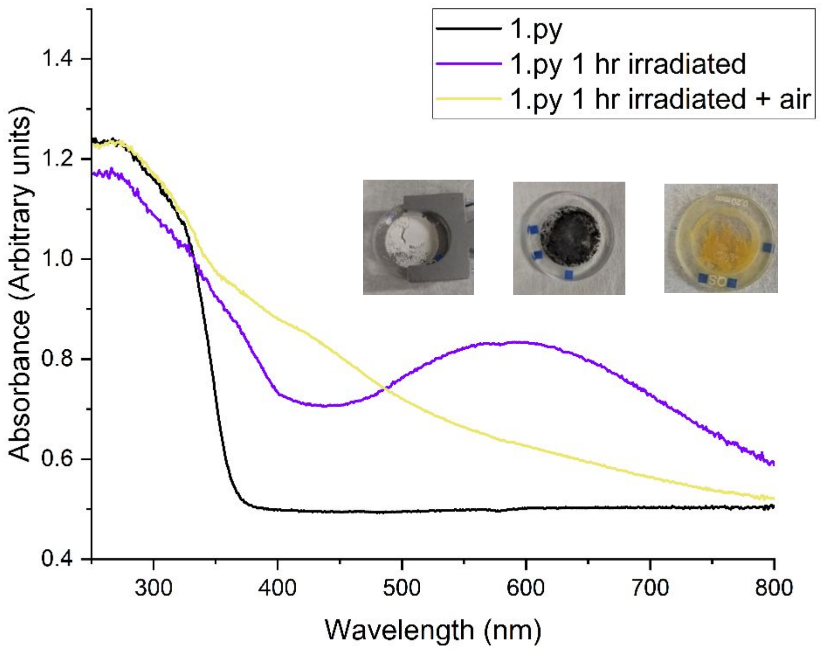

Figure S5. UV-Vis absorption plot of **1.py** as a powder as synthesised (black), after irradiation with 302 nm lamp for 1 hour (purple) and after subsequent oxidation with air (yellow). Inset in the same sequence shows images of the powders of **1.py** in the quartz cells. Note that the baseline is shifted upwards relative to the reference sample due to some light leakage associated with the use of an airtight sample holder in the diffuse reflectance experiment.

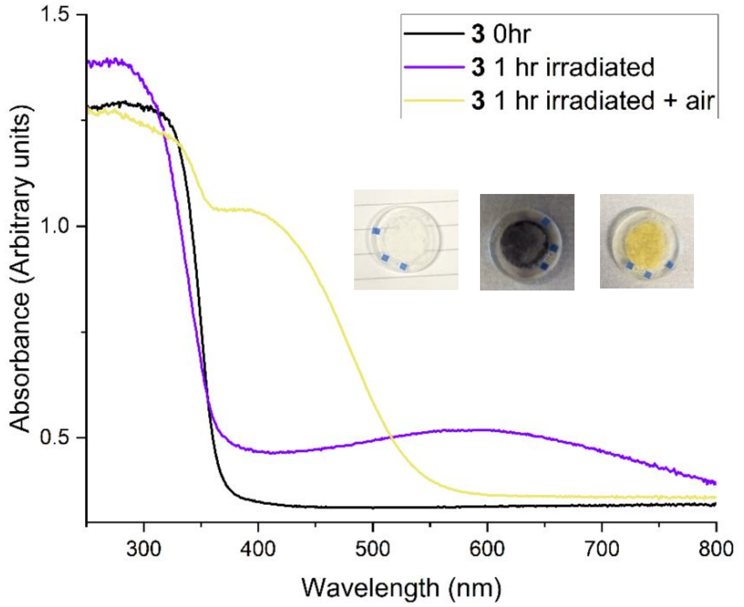

Figure S6. UV-Vis absorption plot of powders of **3** as synthesised (black), after irradiation with 302 nm lamp for 1 hour (purple) and after subsequent oxidation with air (yellow). Inset in the same sequence images of the powders of **3** in the quartz cells. Note that the baseline is shifted upwards relative to the reference sample due to some light leakage associated with the use of an airtight sample holder in the diffuse reflectance experiment.

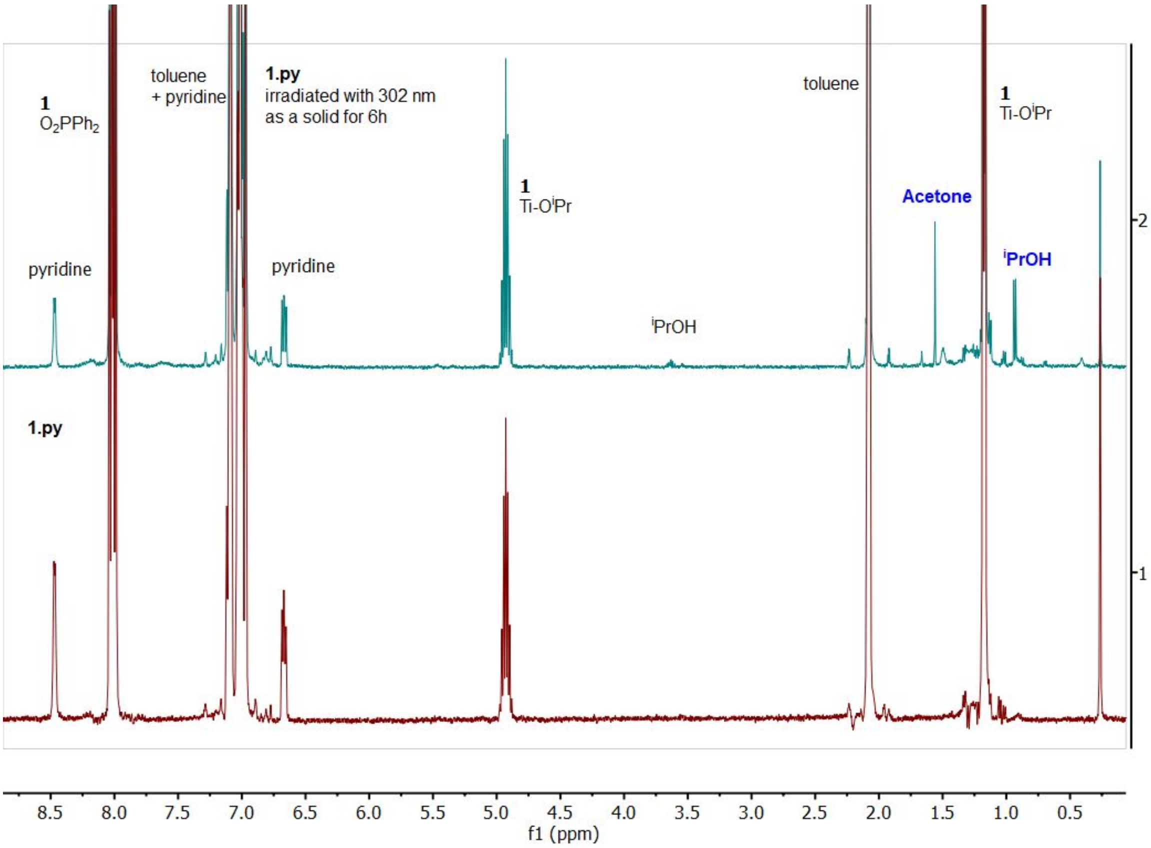

Figure S7. ^{1}H NMR spectra of **1.py** (bottom) and **1.py** after irradiation with 302 nm for 6 h in the solid state (top), both dissolved in d^8-toluene. Analysis of the spectra suggest ~6% of the sample has undergone photoredox reactivity in the solid-state under these conditions with equal amounts of acetone (1.56 ppm) and $^{i}PrOH$ (0.94, 3.63) produced in the process. The small quantity of photoreduced Ti-oxo cluster is not observed, consistent with previous reports and the likelihood of a paramagnetic species.[2] Associated $^{31}P\{^{1}H\}$ NMR spectra remained unchanged, with a single peak observed at 32.47 ppm.

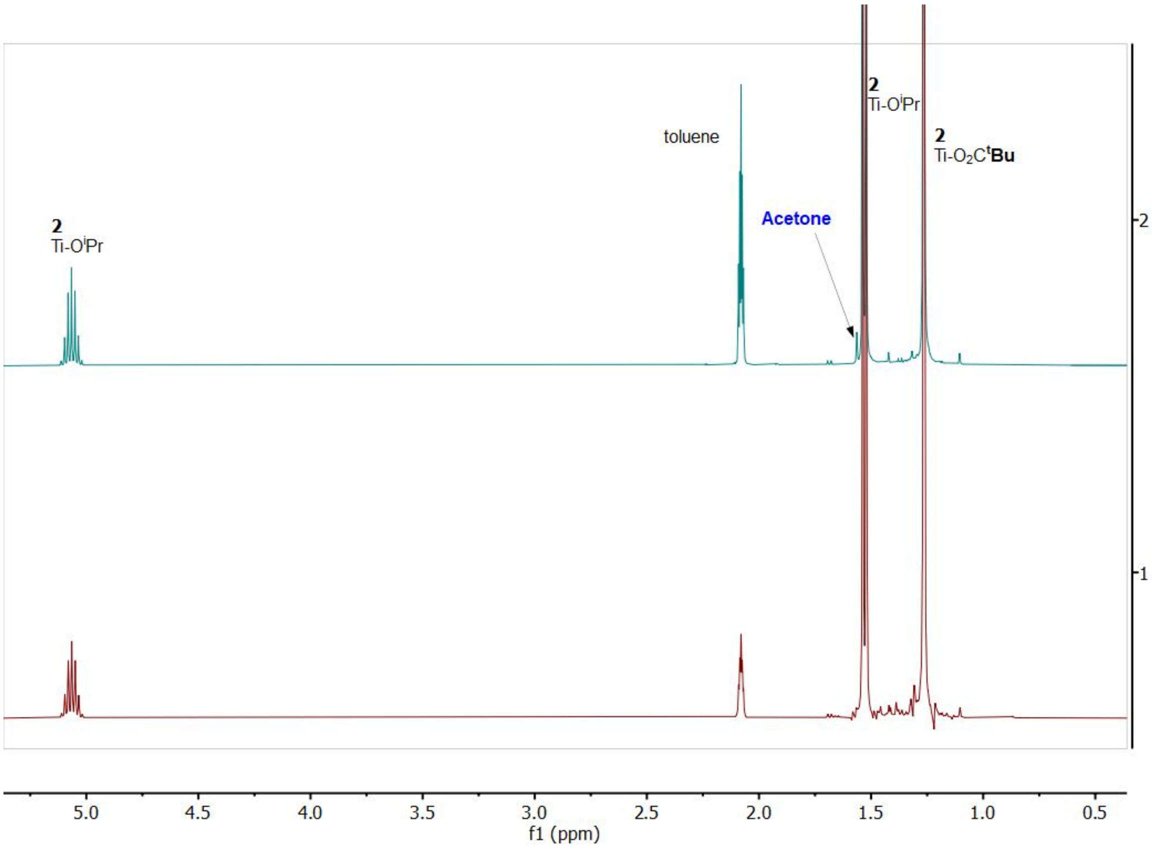

Figure S8. ^{1}H NMR spectra of **2** (bottom) and **2** after irradiation with 302 nm for 6 h in the solid state (top), both dissolved in d^8-toluene. Analysis of the spectra suggest ~9% of the sample has undergone photoredox reactivity in the solid-state under these conditions with acetone (1.56 ppm) produced in the process. The small quantity of photoreduced Ti-oxo cluster is not observed. The photoreduced cluster is expected to coordinate any produced ^{i}PrOH (1 equiv. expected) under these non-coordinating solvent conditions, and therefore free ^{i}PrOH is also not observed. It is noteworthy that the reported spectra of the photoproduct of **2** in the presence of *excess* ^{i}PrOH, $[Ti_6O_6(O^iPr)_4(^iPrOH)_4]$, is similar to **2**, with overlap of signals expected.[8]

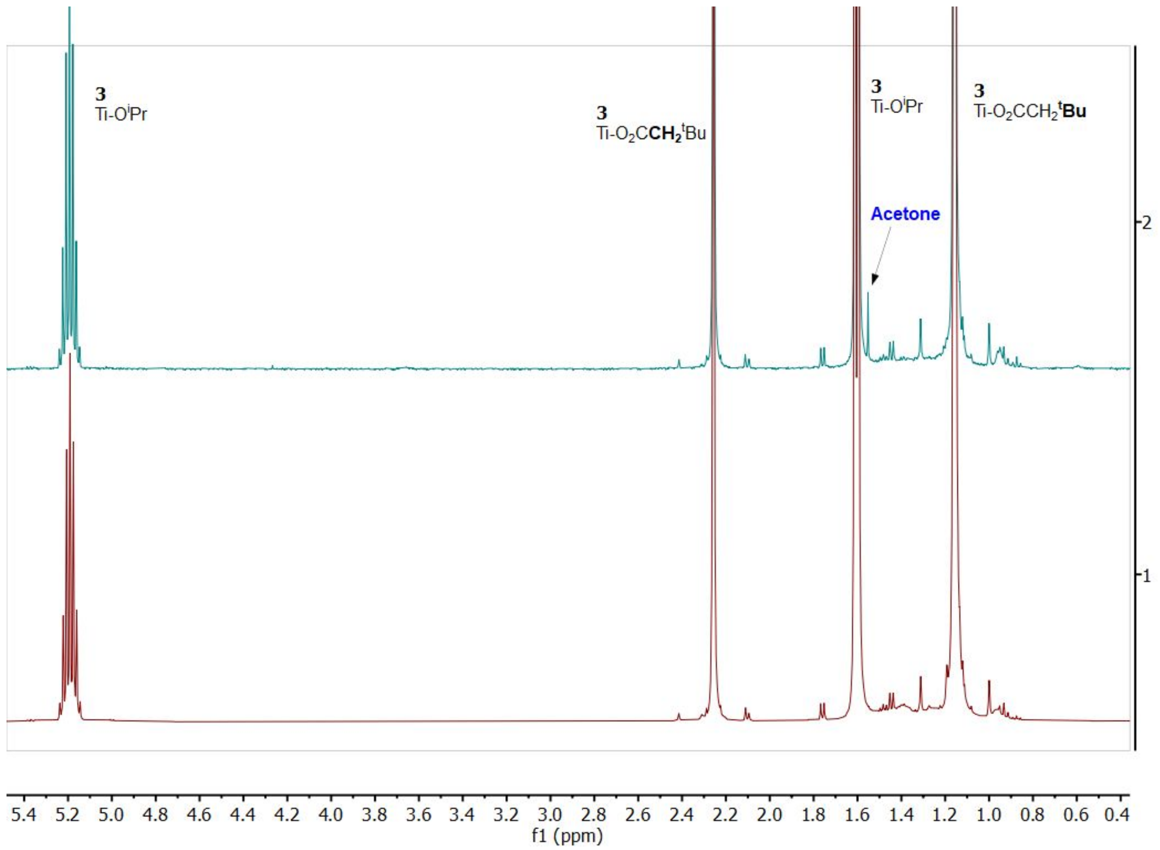

Figure S9. ^{1}H NMR spectra of **3** (bottom) and **3** after irradiation with 302 nm for 6 h in the solid state (top), both dissolved in C_6D_6. Analysis of the spectra suggest ~3% of the sample has undergone photoredox reactivity in the solid-state under these conditions with acetone (1.55 ppm) produced in the process. The small quantity of photoreduced Ti-oxo cluster is not observed. The photoreduced cluster is expected to coordinate any produced ^{i}PrOH (1 equiv. expected) under these non-coordinating solvent conditions, and therefore free ^{i}PrOH is also not observed. It is noteworthy that in related compound **2**, the reported spectra of the photoproduct $[Ti_6O_6(O^iPr)_4(^iPrOH)_4]$ is similar to **2**, with overlap of signals expected.[8]

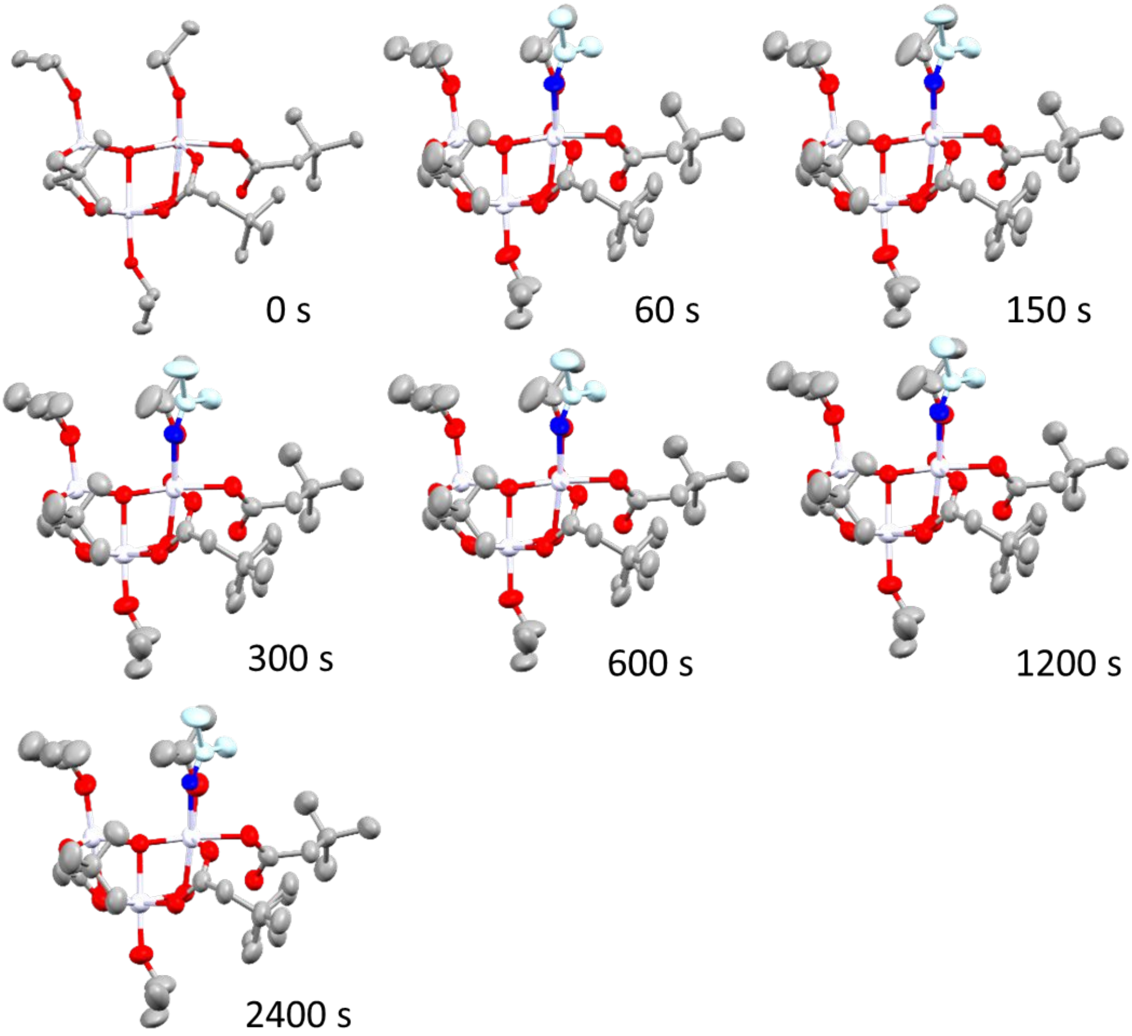

Figure S10. Time series of crystal structures of **3** before and after laser irradiation (labelled by duration of laser irradiation). Displacement ellipsoids drawn at 50 %, H atoms omitted for clarity. Titanium = lilac, oxygen = red, carbon = grey, disordered acetone fragment drawn in blue (oxygen = dark blue, carbon = light blue) only one asymmetric unit of the molecule is drawn.

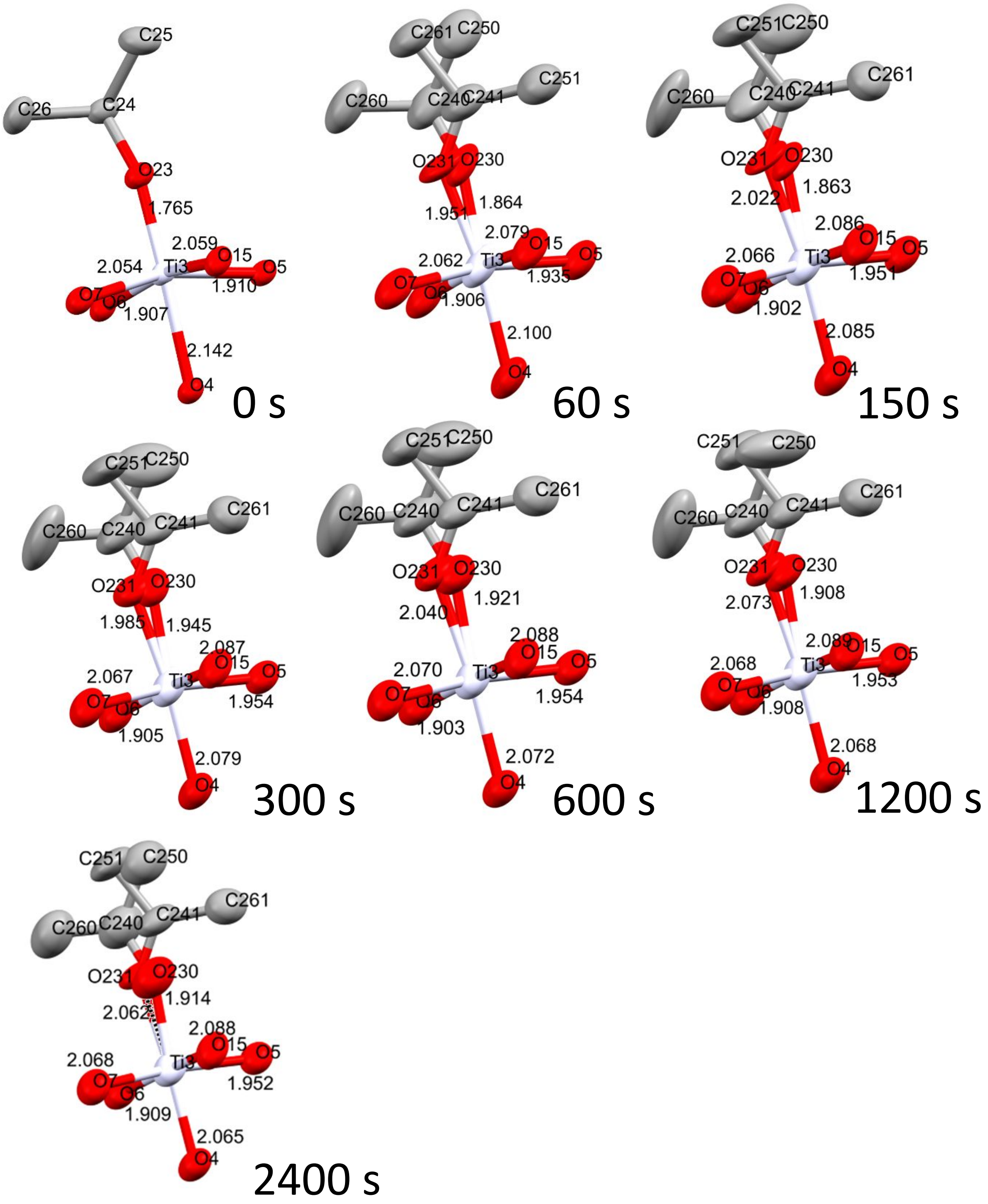

Figure S11. Time series of crystal structures of Ti3 local coordination geometry **3** before and after laser irradiation (labelled by duration of laser irradiation). Displacement ellipsoids drawn at 50 %, H atoms omitted for clarity. Titanium = lilac, oxygen = red, carbon = grey, disordered acetone fragment drawn in same style. Note that in the main text O23, O230, O231, C24, C240, C241 are named O3, O3a, O3b, C3, C3a, C3b respectively.

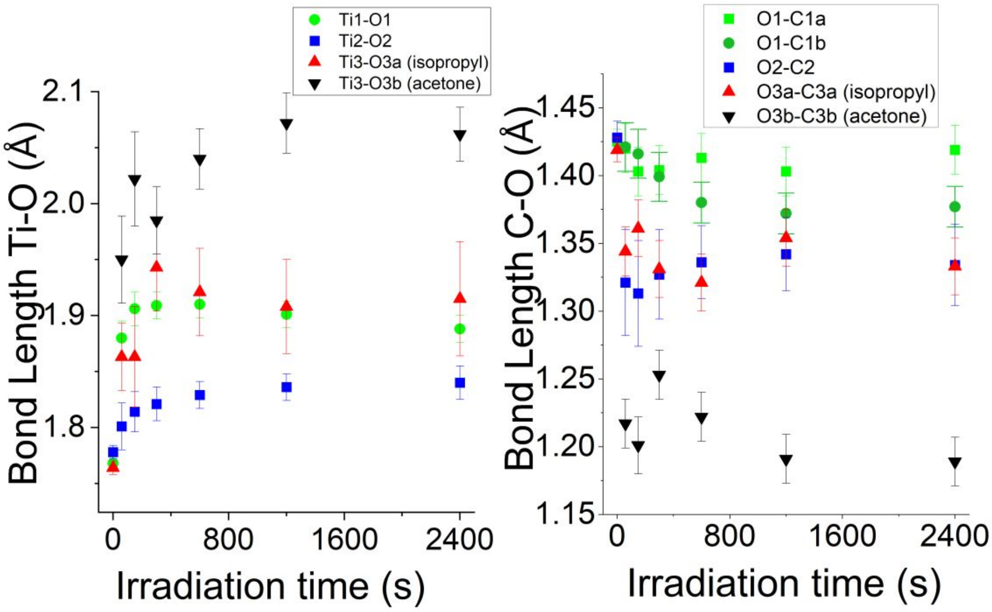

Figure S12. Graphs showing structural parameters calculated from crystal structure models of **3** for each Ti environment over the total irradiation timeline. The Ti3 site is bifurcated into an isopropyl (sp^3 carbon) and acetone (sp^2 carbon) fragment once irradiation is initiated. Error bars drawn at ± 3σ. Left Panel: Ti–O^iPr bond lengths. Right Panel: O–C bond lengths in isopropoxide/acetone ligands.

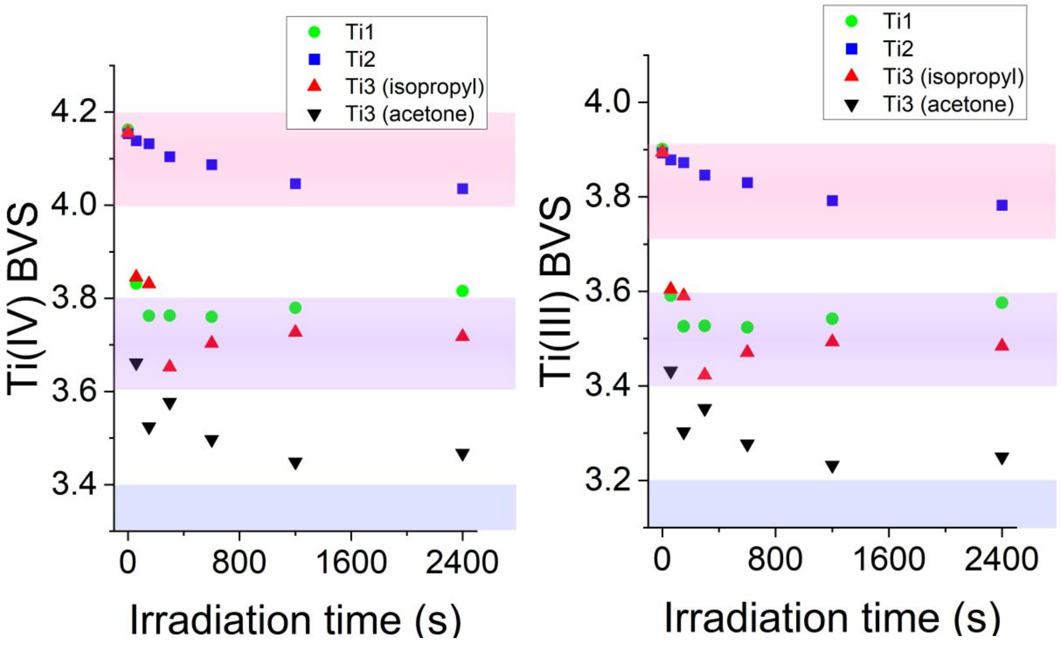

Figure S13. Bond valence sum calculations provide oxidation state information by using the local bond parameters of an atom. The calculation relies on deviation from expected tabulated values e.g. a Ti(4+) site should give a value of ~4 using Ti(4+) parameters, and a Ti(3+) gives ~3 using Ti(3+) parameters.[9] In a delocalised system e.g. with a formal oxidation state of Ti(3.5+), Ti(4+) parameters will give a value of ~3.6 whilst Ti(3+) parameters will return a value of ~3.4.[2, 8] Bond valence sum calculation of **3** for each Ti environment over the total irradiation timeline using expected geometry for Ti(IV), left, or Ti(III), right. Indicating a selective drop in oxidation state with three distinct regions assigned as Ti^{4+} (red shaded), $Ti^{3.5+}$ (purple shaded), and Ti^{3+} (blue shaded) based on reported bond valence sum values from previously reported well-resolved related structures.[2, 8]

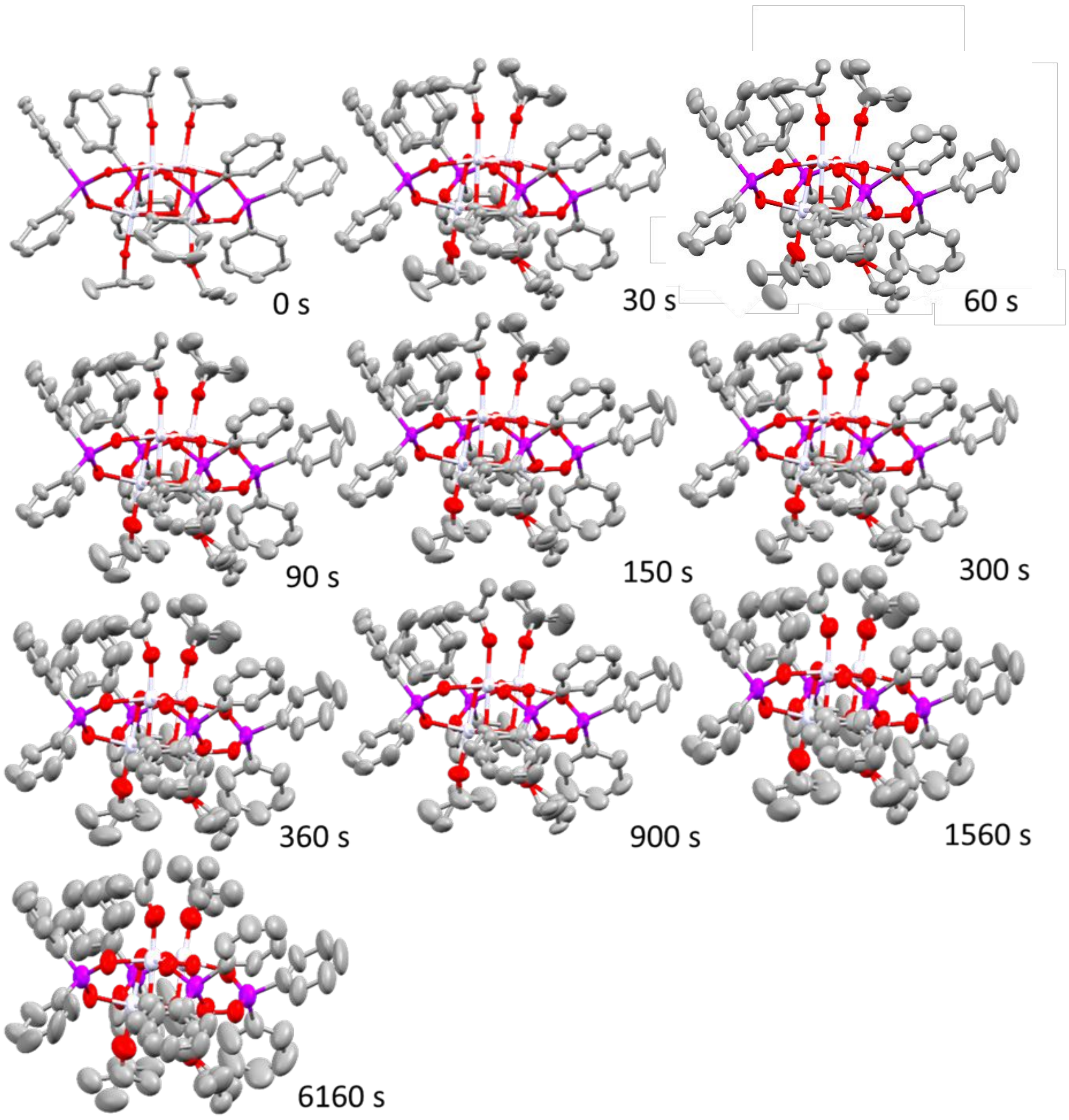

Fig S14. Time series of crystal structures of **1.py** before and after laser irradiation (labelled by duration of laser irradiation). Titanium = lilac, carbon = grey, oxygen = red, phosphorus = purple, displacement ellipsoids drawn at 50%, all disorder shown, H atoms removed for clarity.

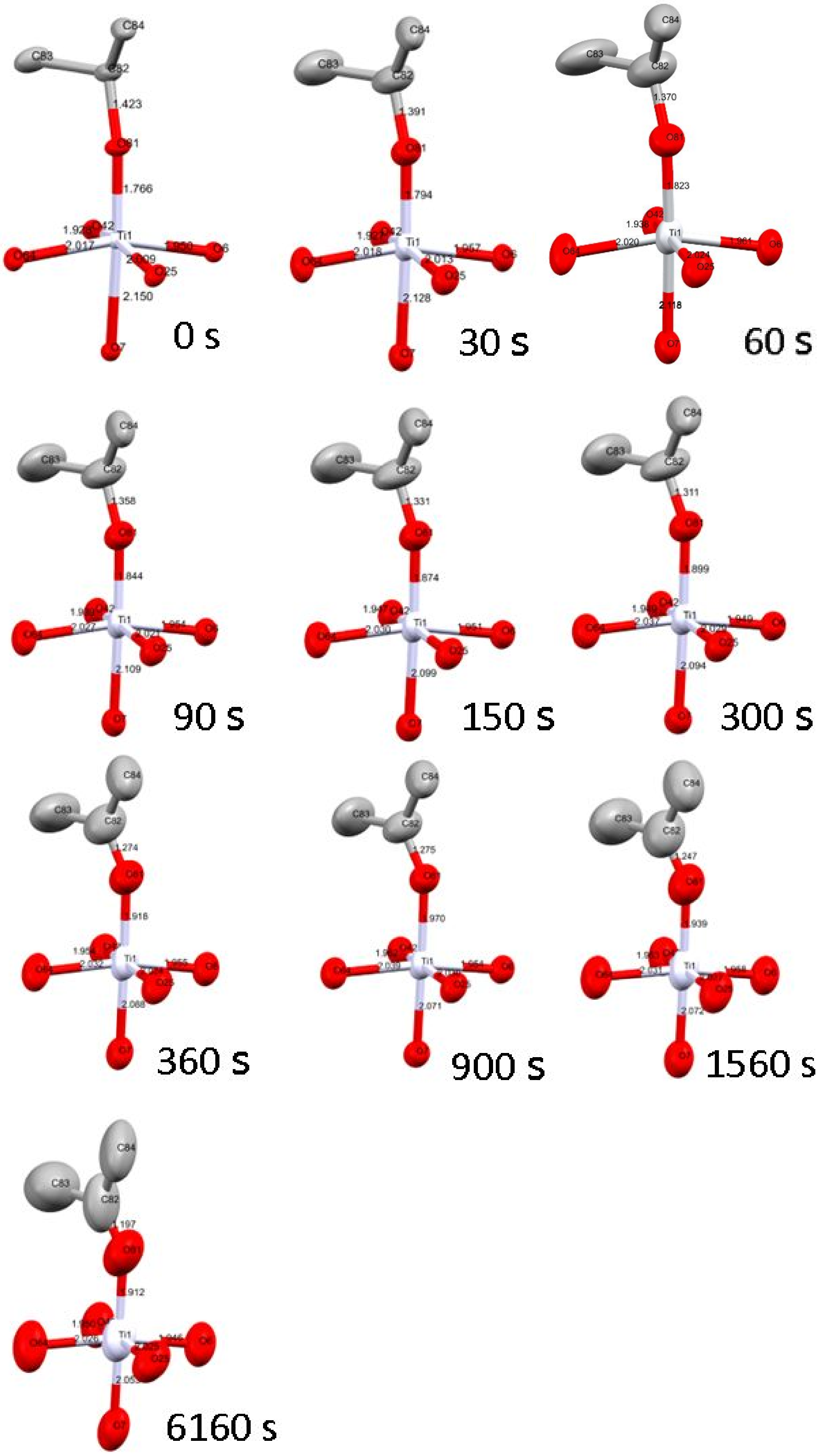

Fig S15. Time series of crystal structures of local coordination in **1.py** before and after laser irradiation (labelled by duration of laser irradiation). Titanium = lilac, carbon = grey, oxygen = red, displacement ellipsoids drawn at 50%, H atoms removed for clarity. Note that in main text O81 and C82 are referred to as O1 and C1 respectively.

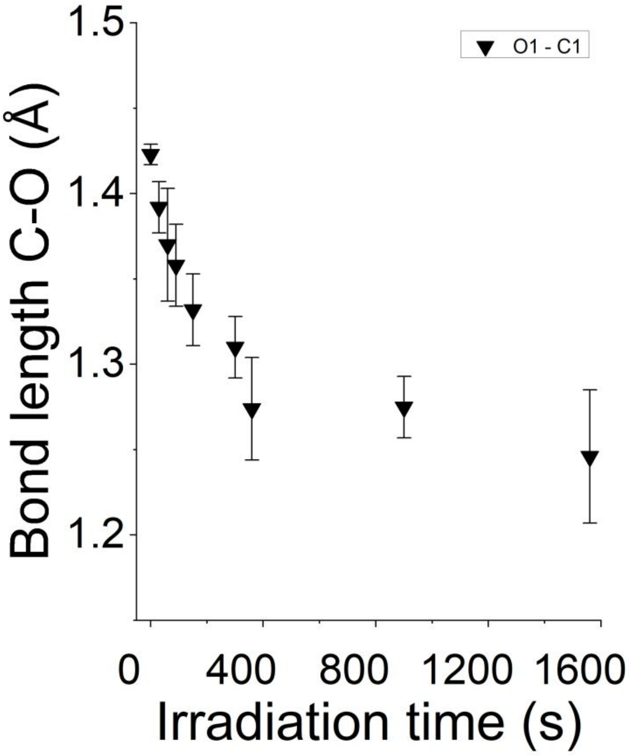

Figure S16. O–C bond lengths in acetone-like fragment that forms at Ti1 on **1.py**. Graph showing structural parameters calculated from crystal structure models of **1.py** for each Ti environment over the total irradiation timeline. Error bars drawn at ± 3σ.

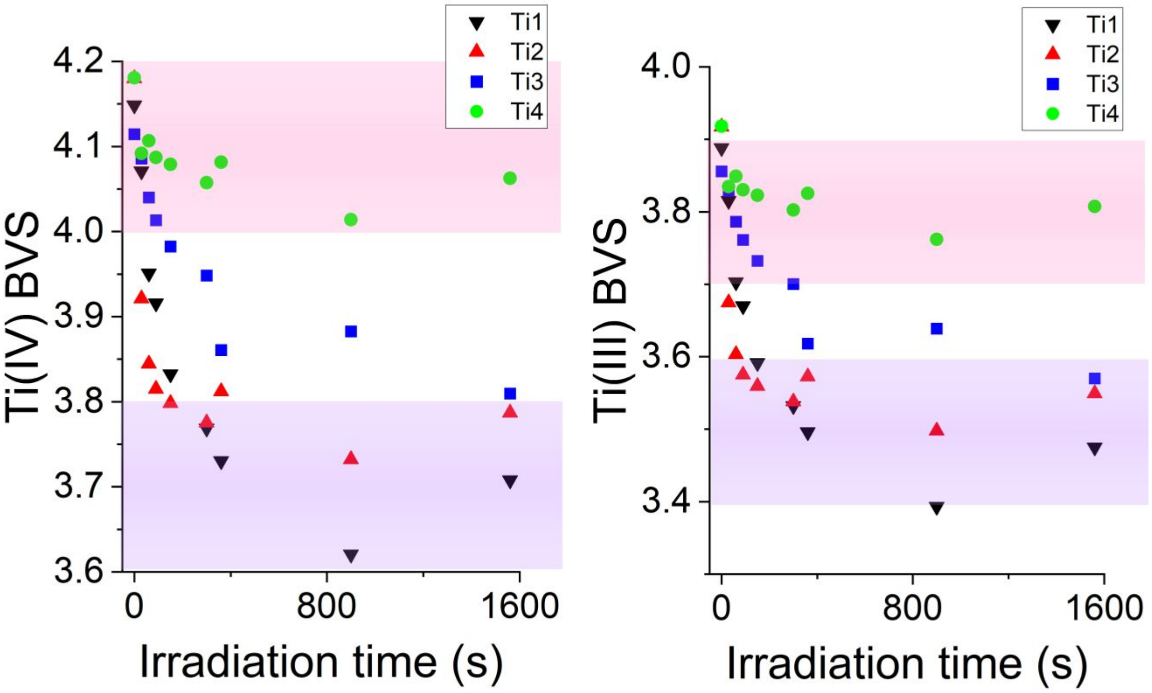

Figure S17. Bond valence sum calculations provide oxidation state information by using the local bond parameters of an atom. The calculation relies on deviation from expected tabulated values e.g. a Ti(4+) site should give a value of ~4 using Ti(4+) parameters, and a Ti(3+) gives ~3 using Ti(3+) parameters.[9] In a delocalised system e.g. with a formal oxidation state of Ti(3.5+), Ti(4+) parameters will give a value of ~3.6 whilst Ti(3+) parameters will return a value of ~3.4.[2, 8] Bond valence sum calculation of **1.py** for each Ti environment over the total irradiation timeline using expected geometry for Ti(IV), left, or Ti(III), right. Indicating a selective drop in oxidation state with two distinct regions assigned as Ti^{4+} (red shaded), $Ti^{3.5+}$ (purple shaded) based on reported bond valence sum values from previously reported well-resolved related structures.[2, 8]

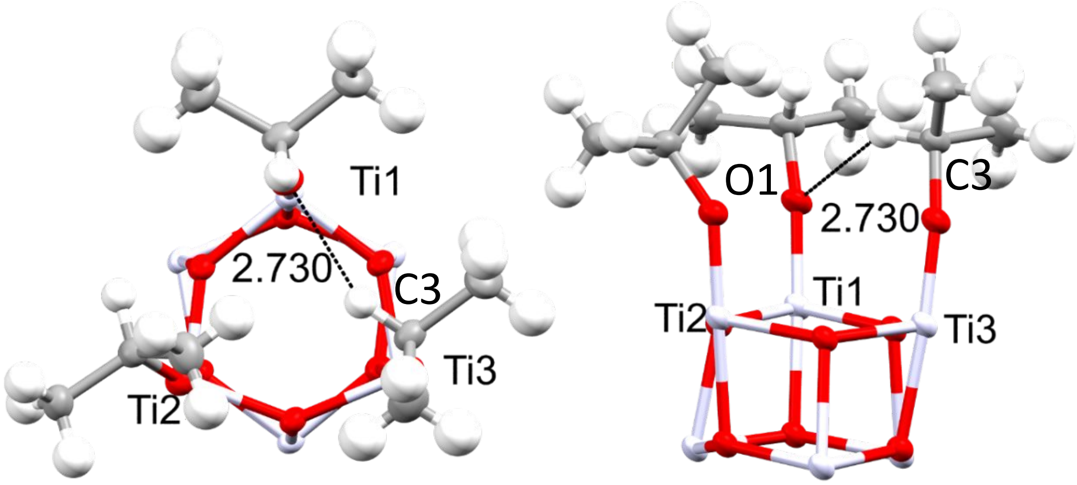

Figure S18. View of the Ti_6O_6 core of **3** with three O^{i}Pr groups shown. The O^{i}Pr groups in **3** have different orientations. The Ti1 and Ti3 O^{i}Pr sites have their Me_2**C–H** bond oriented to point into the centre of the hexagonal Ti_3O_3 face, in contrast, the O^{i}Pr at Ti2 is oriented so that a methyl group points into the hexagonal face. The Ti3 site O^{i}Pr group is oriented so that the a proton points towards the O on a neighbouring group, with a short C3–H···O1 distance of ~2.7 Å (shown in the figure). Other C–H···O distances: C3–H···O2, 3.5 Å; C2–H···O1, 3.5 Å; C2–H···O3, 5.1 Å; C1–H···O2, 4.2 Å; C1–H···O3, 4.3 Å.

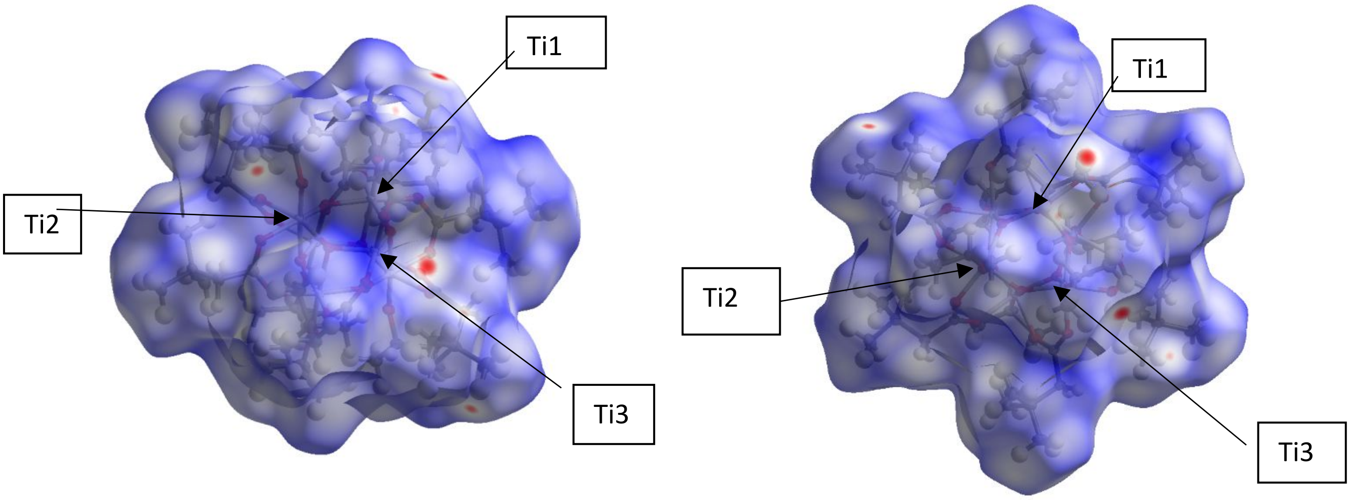

Figure S19. Molecular Hirshfeld d_{norm} surfaces of **3** showing the relative Van der Waals contact distance (Blue, maximum +1.63 Å, white ~0 Å, red -0.12 Å). Crystal Explorer 21.5 was used to construct the molecular Hirshfeld surfaces in the crystal structure of **3**, red indicates intermolecular contacts shorter than the van der Waals radii, white indicates intermolecular distance near the sum of Van der Waals radii with zero d_{norm} value, and blue indicates contact distances longer than the sum of van der Waals radii.[10] In **3** most of the isopropoxide ligand environments appear to have long nearest contacts (shown in blue in the plot), suggesting there is accessible space for some molecular rearrangement. Notably the Ti1 isopoproxide fragment has one close contact, which may limit its mobility and therefore reactivity. In contrast, Ti2 and Ti3 isopropoxides have similar cavity space surrounding them.

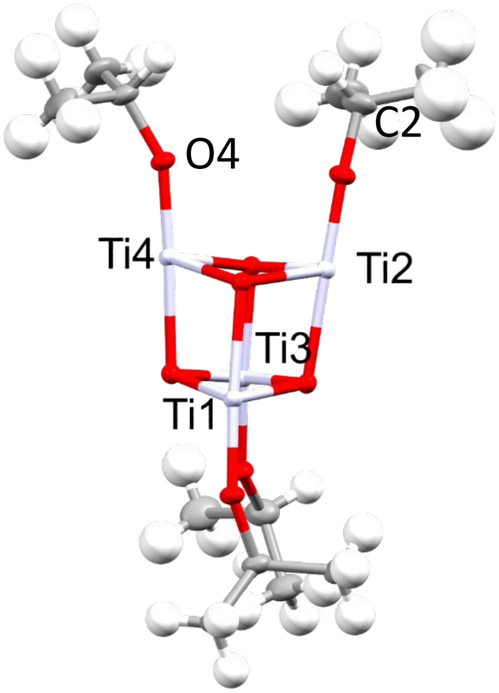

Figure S20. View of **1.py** showing orientation of Ti2 O^{i}Pr site. C2–H···O4 distance ~3.5 Å ($O_2C^{t}Bu$ ligands and pyridine omitted for clarity). N.B. Ti4 and Ti3 sites, poorly oriented for H-atom transfer, have C–H···O distances of 4.0 Å and 5.1 Å respectively.

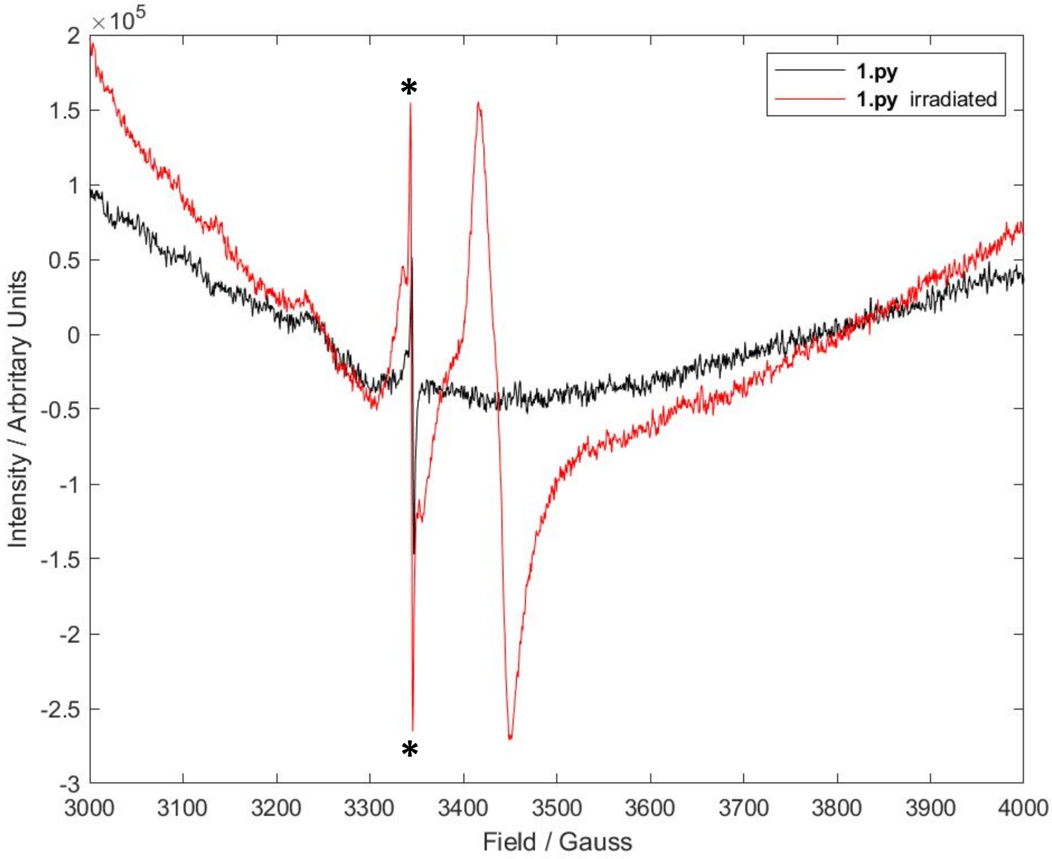

Figure S21. X-band EPR spectra of **1.py** at room-temperature (292 K) before (black) and after (red) irradiating the quartz tube with 302 nm UV light for 1 hour collected with the same spectrometer settings (mw power, time constant, conversion time, modulation frequency, and modulation amplitude). There is a small paramagnetic signal at g =2.00 (marked with *) in the unirradiated sample that is attributed to an organic contaminant in or on the EPR tube, a second EPR tube containing the same batch of **1.py** (see Fig S26) was measured and did not show this impurity signal. After irradiation a new paramagnetic signal is observed at g = 1.95 consistent with a titanium based electronic state.[2, 11]

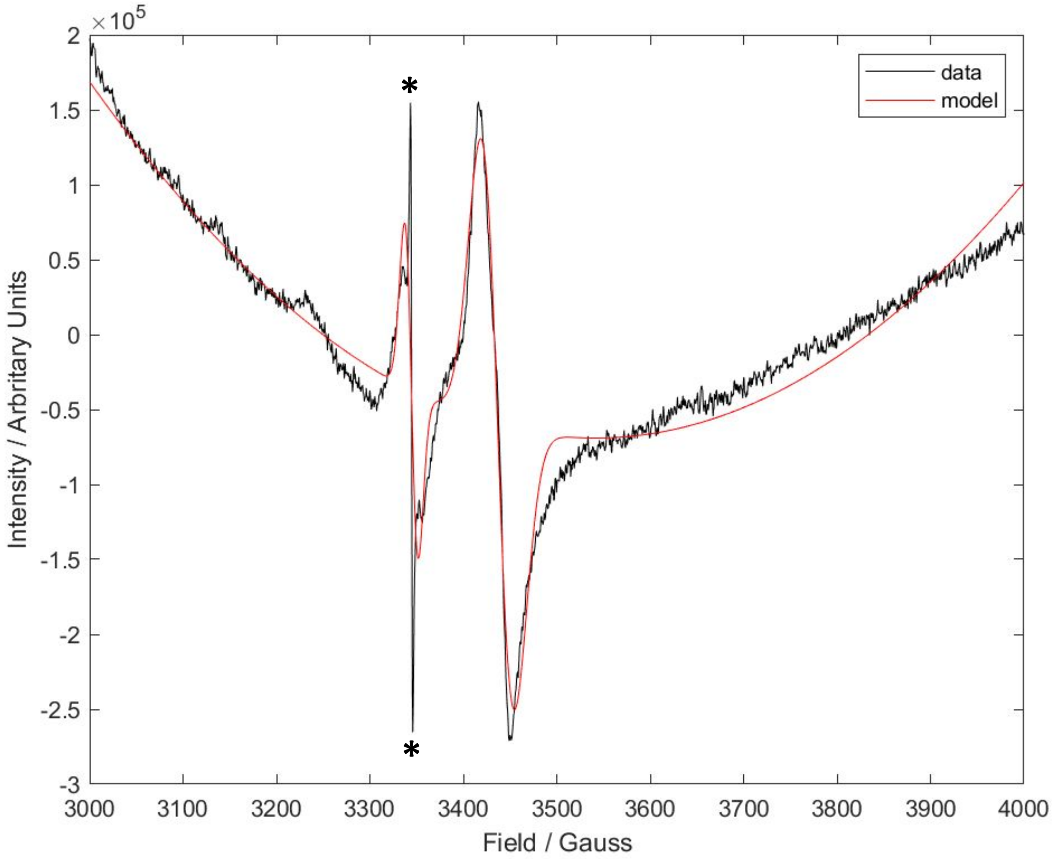

Figure S22. X-band spectrum (black) and simulated (red) spectrum of **1.py** at room-temperature (292 K) after (red) irradiating the quartz tube with 302 nm UV light for 1 hour. The irradiated sample was a purple grey colour. Simulated spectra models 2 isotropic spin systems with relative concentrations of 1:10.5 in order to account for minor impurity signal (marked with *). Major signal; g = 1.948 and gaussian linewidth 42.2. Impurity signal; g = 2.002 and gaussian linewidth of 17.2 G. The major component at 1.948 is consistent with previously reported paramagnetic Ti-oxo clusters.[2, 11]

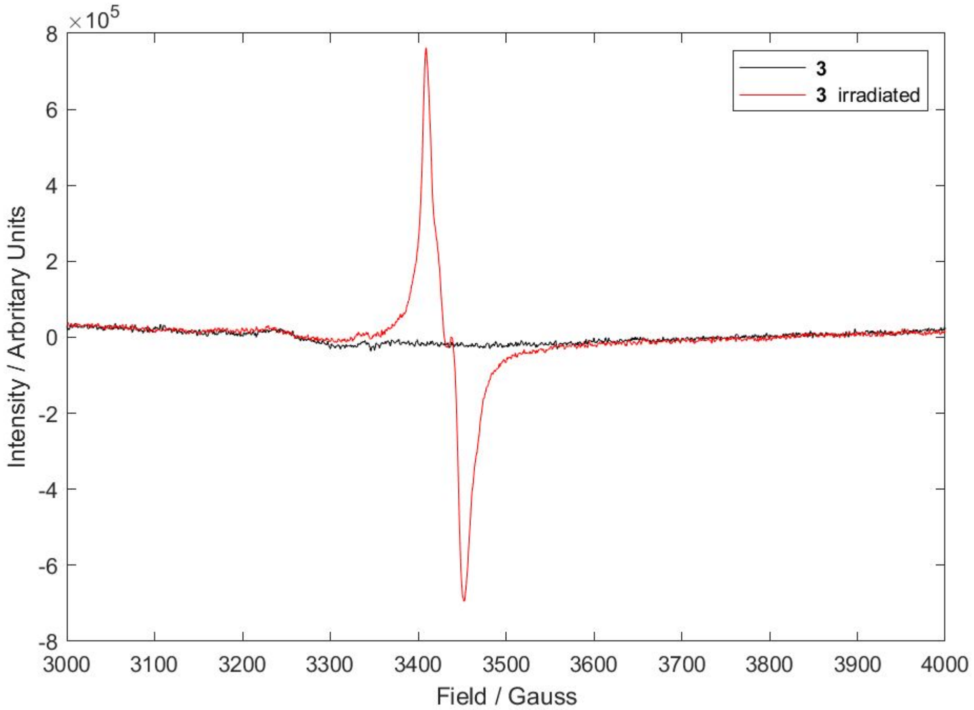

Figure S23. X-band spectra of **3** at room-temperature (292 K) before (black) and after irradiating the quartz tube with 302 nm UV light for 1 hour in air (red). The irradiated sample was grey/purple in colour. Spectra collected with the same spectrometer settings (mw power, time constant, conversion time, modulation frequency, and modulation amplitude). After irradiation a new paramagnetic signal is observed at g = 1.951 consistent with a titanium-based spin system.[2, 11]

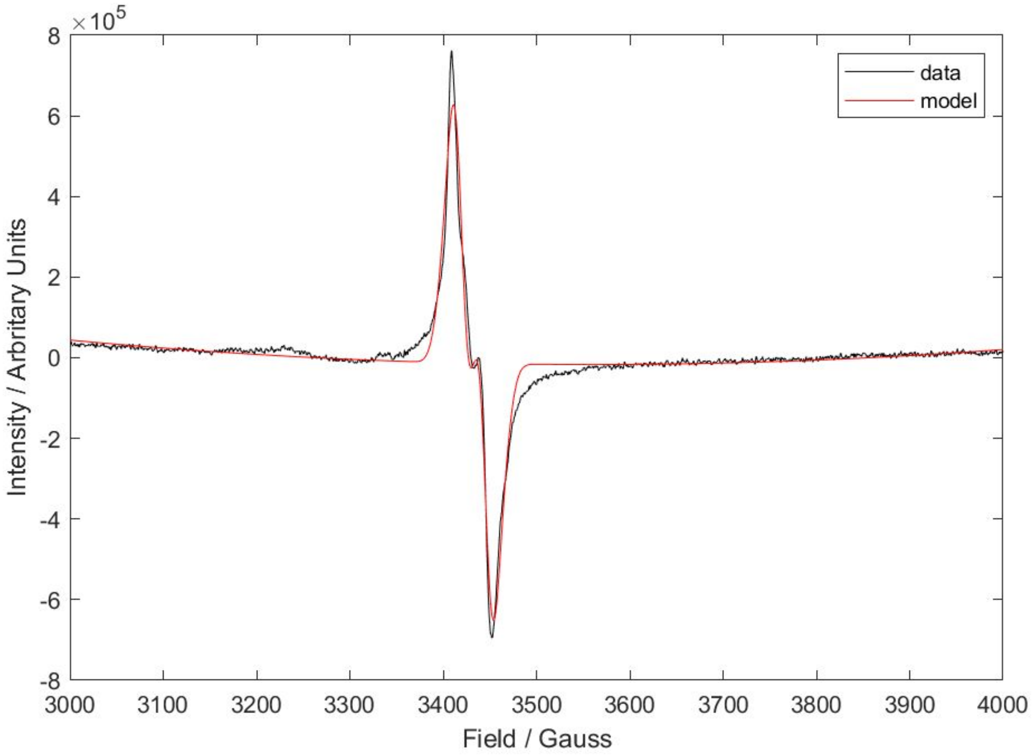

Figure S24. X-band spectrum (black) and simulated (red) spectrum of **3** at room-temperature (292 K) after (red) irradiating the quartz tube with 302 nm UV light for 1 hour. Simulated spectra models an isotropic triplet spin systems with g = 1.951, gaussian linewidth of 21G, and D = 87.1 MHz.[2]

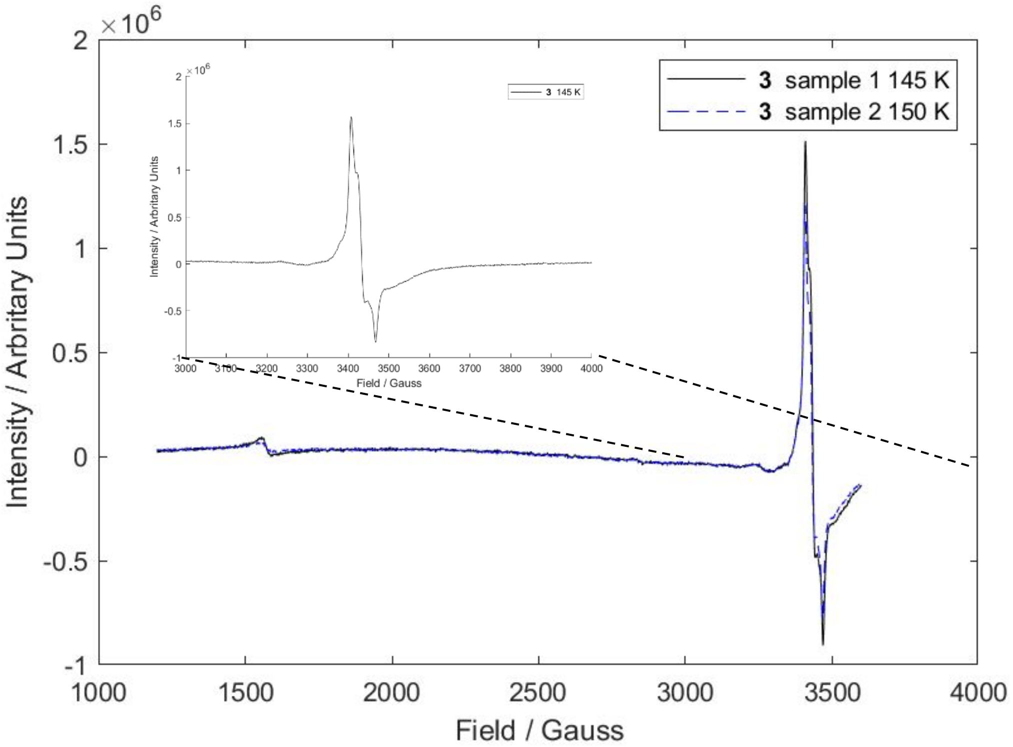

Figure S25. X-band spectrum of two separately prepared samples of **3** at 145 K and 150 K after irradiating the quartz tube with 302 nm UV light at room temperature for 1 hour. Main spectrum shows g ~2 peak and also low-field transition at g ~4, characteristic of the presence of a triplet state. Inset with spectrum centred on main transition.[2, 12]

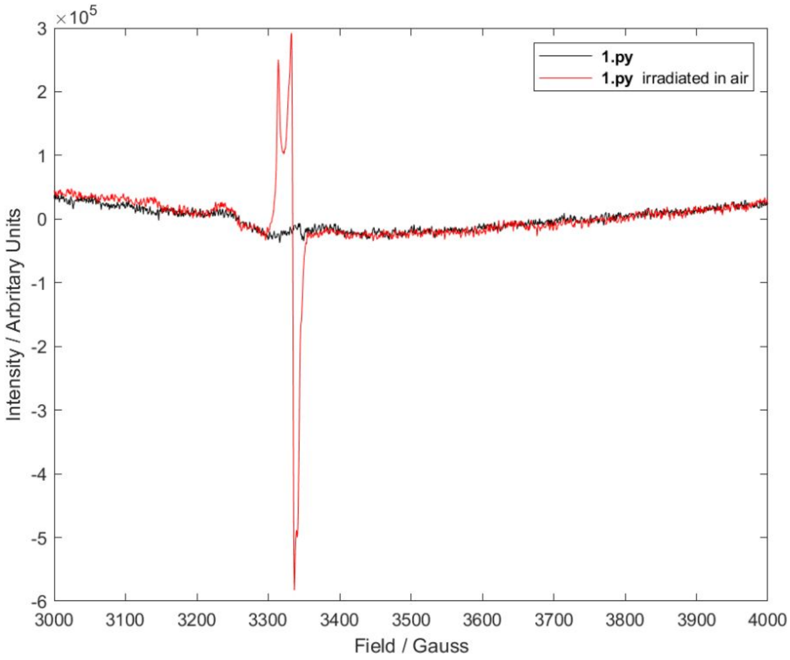

Figure S26. X-band spectra of **1.py** at room-temperature (292 K) before (black) and after (red) irradiating the quartz tube with 302 nm UV light for 1 hour in air. The irradiated sample rapidly oxidised under air and was yellow in colour. Spectra collected with the same spectrometer settings (mw power, time constant, conversion time, modulation frequency, and modulation amplitude). After irradiation a new paramagnetic signal is observed at $g_{\perp}$ = 2.007 and g = 2.021 consistent with formation of a superoxide species.[2]

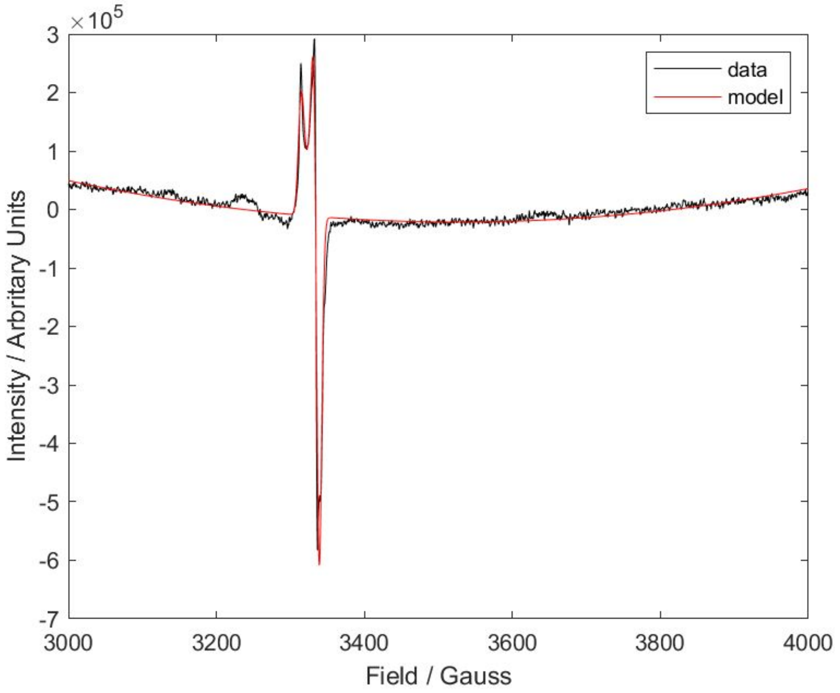

Figure S27. X-band spectrum (black) and simulated (red) spectrum of **1.py** at room-temperature (292 K) after (red) irradiating the quartz tube with 302 nm UV light for 1 hour in the presence of air as an oxidant. The irradiated and oxidised sample was a yellow colour in contrast to the purple colour observed without air present. Simulated spectra models an axial spin systems with $g_{\perp}$ = 2.007 and g = 2.021 and gaussian linewidth of 9.1G.

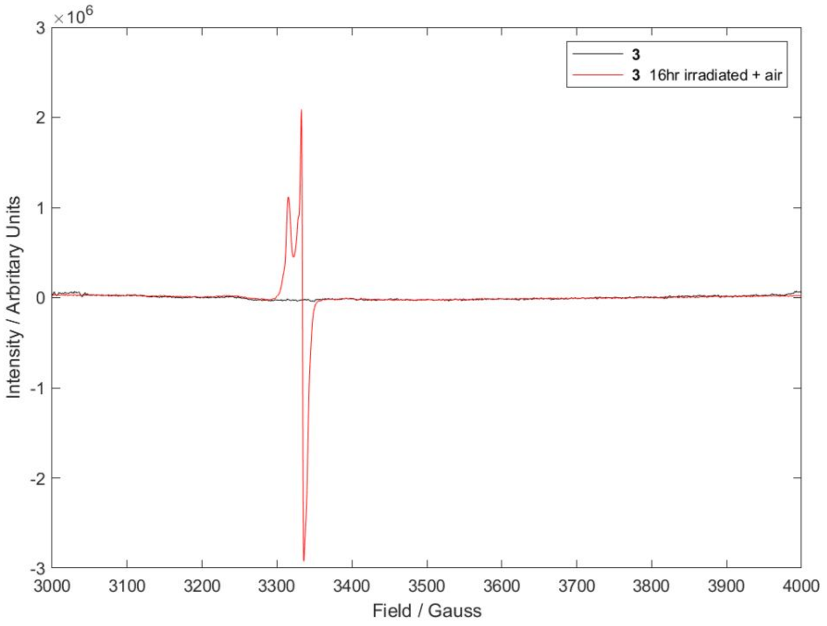

Figure S28. X-band spectra of **3** at room-temperature (292 K) before (black) and after (red) irradiating the quartz tube with 302 nm UV light for 16 hours in air. The irradiated sample oxidised under air and was yellow in colour. Spectra collected with the same spectrometer settings (mw power, time constant, conversion time, modulation frequency, and modulation amplitude). After irradiation a new paramagnetic signal is observed at $g_{\perp}$ = 2.007 and g = 2.020 consistent with formation of a superoxide species.[2]

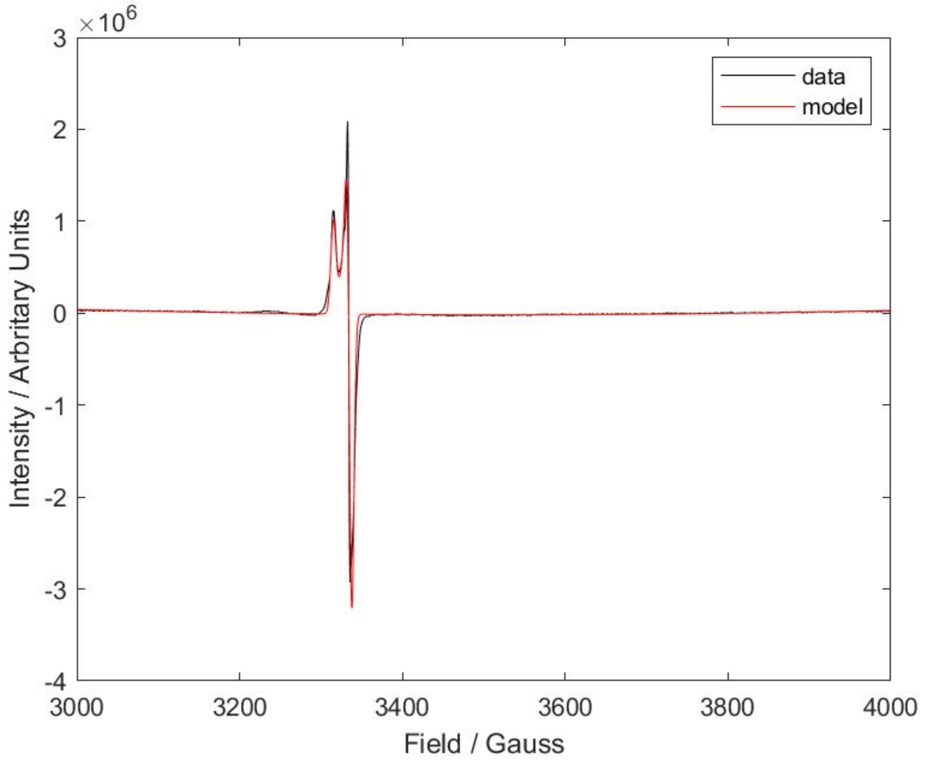

Figure S29. X-band spectrum (black) and simulated (red) spectrum of **3** at room-temperature (292 K) after (red) irradiating the quartz tube with 302 nm UV light for 16 hours in air. Simulated spectra models axial spin systems with $g_{\perp}$ = 2.007 and g = 2.020 and, gaussian linewidth of 7.2G.

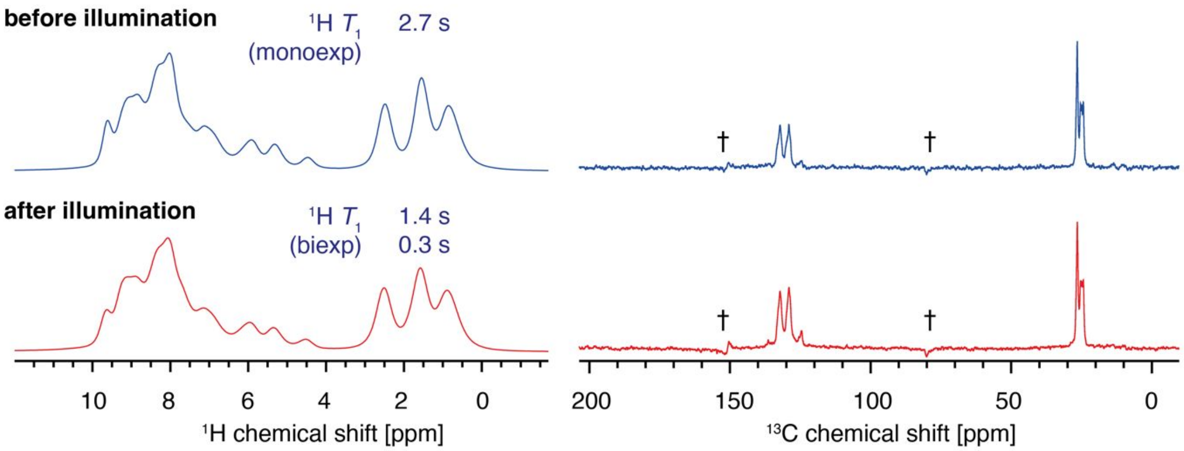

Figure S30. ^{1}H (quantitative) and ^{13}C echo-detected MAS NMR spectra of **1.py** before and after illumination recorded at 23 T, 30 kHz MAS and 298 K. † indicates an experimental artefact.

Supporting Note 1. Exploration of co-crystallised solvent molecules

The photoreactivity of several crystalline forms of compound **1** containing different co-solvent molecules were explored as part of this project. These crystals were prepared by recrystallisation of **1.tol** from different solvents as described below.

1.DMSO

1.DMSO has been reported previously from the synthesis of **1** in DMSO.[13]

Alternatively **1.DMSO** can be prepared from **1.tol** in DMSO. 30 mg (0.021 mmol) of **1.tol** was dissolved in minimal hot DMSO (75 °C) and upon cooling slowly to room temperature crystals of **1.DMSO** were formed.

1.^{i}PrOH

1.^{i}PrOH has been reported previously from the synthesis of **1** in ^{i}PrOH.[14]

Alernatively, **1.^{i}PrOH** can be prepared by suspending **1.tol** in ^{i}PrOH in a sealed Teflon lined autoclave. The Flask was heated to 120°C and then allowed to cool slowly to yield crystals.

1.THF

30 mg (0.021 mmol) of **1.tol** was dissolved in minimal THF, and this solution was layered with pentane to yield crystals of **1.THF.**

1.2MeCN

30 mg (0.021 mmol) of **1.tol** was dissolved in minimal MeCN. After leaving to stand crystals of **1.2MeCN** formed.

1 (from Et_2O)

1.tol (30 mg, 0.021 mmol) was partially dissolved in hot diethyl ether. The resulting solution was decanted from the remaining solid and from that solution grew crystals of **1** without co-solvent.

1.tol,[2] **1.py**, **1.DMSO**,[13] **1.^{i}PrOH**,[14] **1.THF** (tol = toluene, py = pyridine) adopt a similar crystal structure (space group = $P2_1/c$). In contrast, **1.2MeCN**, with two solvent molecules, adopts a different structure (space group = $P2_1/m$). If crystals of **1** are prepared from a solution of Et_2O then a crystalline form is found without a solvent molecule (space group = P-1). No major changes are noted in the structure of cluster **1** in the differing crystal forms. These compounds were ground to fine powders and exposed to UV irradiation under inert atmosphere, using a Analytik Jena UVLM-26 EL series UV lamp (output 302 nm, approx. 3 mW/cm2 @ 1 cm, 6 W power).

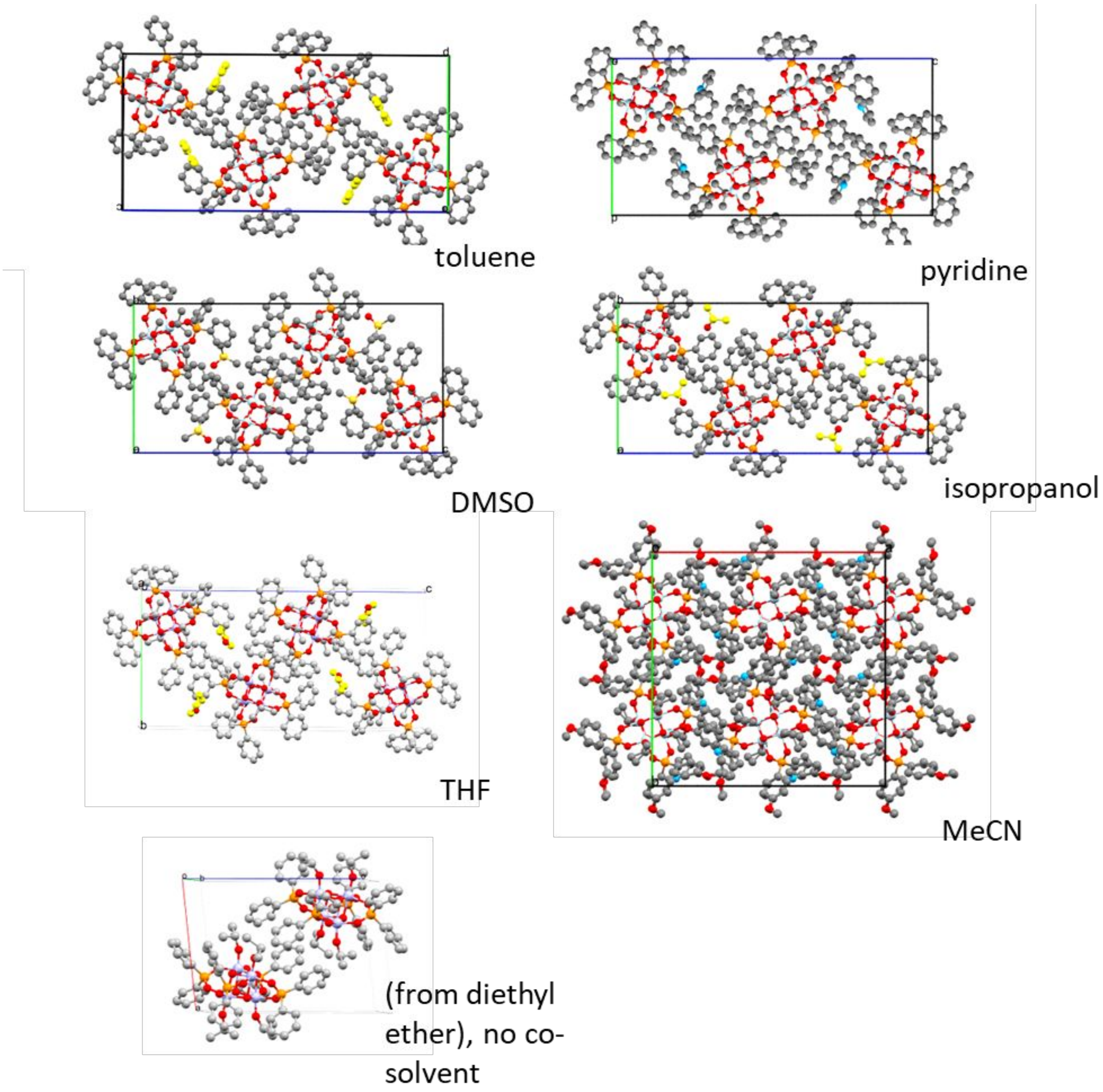

Figure Supporting Note 1. Unit cells of **1** when co-crystallised with different solvent molecules. Toluene, THF, isopropanol carbons highlighted in yellow for clarity.

1.py undergoes a notable white to purple colour change under the UV lamp, whilst the other powders show varying degrees of colour change (Table S1). Note that coordination of pyridine to photoreduced Ti-oxo clusters has been reported to result in a strong purple colour due to metal to (pyridine) ligand charge transfer processes, therefore, the stronger colour found from **1.py** does not necessarily signify a greater photoconversion.[2, 8] In the single crystal phase **1.py** changes colour from colourless to blue under UV light (at 100 K), with evidence that co-crystallised pyridine molecules do not coordinate to the reduced Ti-oxo cluster. In the higher surface area powder phase, under room temperature conditions, the purple colouration may indicate that some ligand exchange to allow pyridine coordination is possible.

In most cases the photoconversion of powders is low (see Table S1) after several hours under the low-power UV lamp, with most photoreactivity expected to occur on the surface of the powders. Without a more careful analysis of the surface areas of these different powders, detailed analysis of the effect of co-solvents on the rate of photoreactivity in the solid-state is unclear. It is possible that the influence of co-crystallised solvent may enact topochemical changes which may cause changes in reaction site selectivity, however, detailed study was beyond the scope of this study.[15]

Irradiated samples (room temperature) of powdered **1.py** were analysed by FTIR spectroscopy within a N_2 filled glovebox. No clear signal for acetone could be detected in this case, we anticipate that the small percentages of photochemical conversion, coupled with the volatility of acetone during room temperature irradiation are most likely to account for this. N.B. acetone formation was confirmed by NMR spectroscopic analysis of the irradiated powder.

Supporting Note 2. Evaluation of X-ray beam damage

Intense X-ray irradiation can induce beam damage, leading to reduction of diffraction quality or even chemical reactivity within a crystal. Experiments show that the unirradiated dataset of **1.py** ('crystal 1', t = 0 s) exhibits different structural metrics to the first data collection of **1.py** 'crystal 2' which was collected after 30s UV irradiation, Figs. S13-17. Different structural metrics are also found for subsequent datasets which have received the same dose of X-rays but different time periods under the laser. This confirms that photochemical transformation under laser UV light is the dominant chemical process occurring in these crystals over the timelines recorded. However, we are currently examining whether X-ray induced redox processes may occur over longer periods of X-ray irradiation in the absence of UV light, and we hope to communicate these findings in the future.

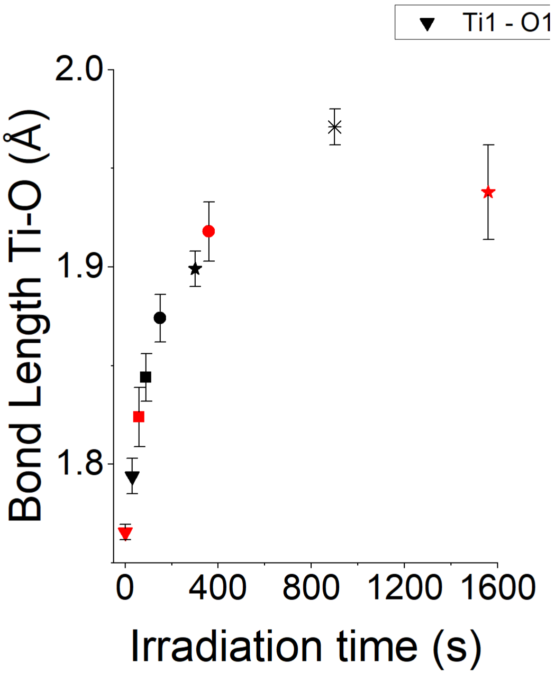

Figure Supporting Note 2. Graph showing Ti(1)–O^iPr bond lengths from crystal structure models of **1.py** for each Ti environment. Error bars drawn at ± 3σ. Crystal 1 data points in red, crystal 2 data points in black. Triangle = exposed to X-rays for one data collection; square = exposed to X-rays for two data collections; circle = exposed to X-rays for three data collections; star = exposed to X-rays for four data collections; asterisk = exposed to X-rays for five data collections.

Crystallography table of new crystal structures

Compound	1.py	1.THF	1.2MeCN	1
CCDC No.	2335755	2335754	2335753	2335752
X-ray source	synchrotron	Cu K-α	Cu K-α	Cu K-α
wavelength [Å]	0.4859	1.54180	1.54180	1.54180
Formula	$Ti_4O_{16}P_4C_{65}N_1H_{73}$	$Ti_4O_{17}P_4C_{64}H_{76}$	$Ti_4O_{16}P_4C_{64}N_2H_{74}$	$Ti_4O_{16}P_4C_{60}H_{68}$
M	1439.79	1432.79	1442.78	1360.69
Crystal System	monoclinic	monoclinic	monoclinic	triclinic
Space Group	P 2_1/c	P 2_1/c	P 2_1/m	P -1
T [K]	100.0(1)	100	100	100
a [Å]	12.431600(10)	12.41220(10)	13.19780(10)	12.3786(2)
b [Å]	16.173700(10)	16.26470(10)	21.39920(10)	16.3022(2)
c [Å]	33.497704(10)	33.37440(10)	13.40320(10)	16.4792(2)
α [deg]	90	90	90	89.3081(10)
β [deg]	96.77	96.6769(4)	113.5334(11)	84.9296(12)
γ [deg]	90	90	90	78.8170(13)
V [$Å^{-3}$]	6688.294(7)	6691.95(7)	3470.53(5)	3249.53(8)
Z	4	4	4	2
θ range [deg]	1.128 - 27.951	2.666 - 77.318	3.597 - 79.819	3.654 - 77.732
Reflections collected	36486	288640	53863	115087
R int	0.108	0.071	0.039	0.095
No. of data/restr/par	18843/0/811	13595/108/829	7664/106/445	13013/1140/1033
R1 [I>2σ(I)]	0.0402	0.0311	0.0399	0.0682
wR2 [all data]	0.1013	0.0832	0.1129	0.1992
GoF	1.0285	1.0003	0.9415	1.0058
Largest diff. pk and hole [eÅ-3]	1.11 & -0.71	0.60 & -0.47	1.48 & -0.75	1.29 & -0.89

Crystallography table of irradiated samples over time

Compound	1.py	1.py	1.py	1.py	1.py
**Crystal**	Crystal 1	Crystal 1	Crystal 1	Crystal 1	Crystal 1
**Irradiation Time [s]**	0	60	360	1560	6160
**CCDC No.**	2335755	-	-	-	-
**X-ray source**	synchrotron	synchrotron	synchrotron	synchrotron	synchrotron
**wavelength [Å]**	0.4859	0.4859	0.4859	0.4859	0.4859
**Formula†**	$Ti_4O_{16}P_4C_{65}N_1H_{73}$	$Ti_4O_{16}P_4C_{65}N_1H_{71}$	$Ti_4O_{16}P_4C_{65}N_1H_{70.52}$	$Ti_4O_{16}P_4C_{65}N_1H_{70.46}$	$Ti_4O_{16}P_4C_{65}N_1H_{70}$
**M**	1439.79	1437.77	1437.28	1437.23	1436.76
**Crystal System**	monoclinic	monoclinic	monoclinic	monoclinic	monoclinic
**Space Group**	$P\ 2_1/c$	$P\ 2_1/c$	$P\ 2_1/c$	$P\ 2_1/c$	$P\ 2_1/c$
**T [K]**	100.0(1)	100.0(1)	100.0(1)	100.0(1)	100.0(1)
**a [Å]**	12.431600(10)	12.425000(14)	12.515901(17)	12.56780(2)	12.59530(2)
**b [Å]**	16.173700(10)	16.193201(14)	16.223001(14)	16.225300(14)	16.216501(17)
**c [Å]**	33.497704(10)	33.52170(2)	33.40450(2)	33.35900(2)	33.32330(3)
**α [deg]**	90	90	90	90	90
**β [deg]**	96.767	96.6920(18)	97.383(3)	97.399(3)	97.376(3)
**γ [deg]**	90	90	90	90	90
**V [$Å^{-3}$]**	6688.294(7)	6688.294(7)	6726.40(4)	6745.80(4)	6750.02(5)
**Z**	4	4	4	4	4
**θ range [deg]**	1.128 - 27.951	1.128 - 27.943	1.122 - 27.988	1.117 - 27.912	1.203 - 27.991
**Reflections collected**	36486	35689	35862	35938	36033
**R int**	0.108	0.151	0.144	0.152	0.487
**No. of data/restr/par**	18843/0/811	6618/908/987	5900/908/987	5180/908/987	2547/908/987
**R1 [I>2σ(I)]**	0.0402	0.0699	0.0554	0.0604	0.0617
**wR2 [all data]**	0.1013	0.1827	0.1606	0.1847	0.1843
**GoF**	1.0285	1.0662	1.1523	1.2772	1.3001
**Largest diff. pk and hole [eÅ-3]**	1.11 & -0.71	0.73 & -0.77	0.59 & -0.58	0.39 & - 0.48	0.46 & -0.51

†irradiated structures of irradiated **1.py** should have 73 H atoms, however, due to the disordered acetone/isopropoxide/isopropanol sites it is not always possible to add all H at a sensible geometry, which leads to a deficit of H in the crystallographic model.

Compound	1.py	1.py	1.py	1.py	1.py
**Crystal**	Crystal 2	Crystal 2	Crystal 2	Crystal 2	Crystal 2
**Irradiation Time [s]**	30	90	150	300	900
**CCDC No.**	-	-	-	-	-
**X-ray source**	synchrotron	synchrotron	synchrotron	synchrotron	synchrotron
**wavelength [Å]**	0.4859	0.4859	0.4859	0.4859	0.4859
**Formula†**	$Ti_4O_{16}P_4C_{65}N_1H_{72.26}$	$Ti_4O_{16}P_4C_{65}N_1H_{70\cdot60}$	$Ti_4O_{16}P_4C_{65}N_1H_{70\cdot59}$	$Ti_4O_{16}P_4C_{65}N_1H_{70\cdot57}$	$Ti_4O_{16}P_4C_{65}N_1H_{72}$
**M**	1439.04	1437.37	1437.36	1437.34	1438.78
**Crystal System**	monoclinic	monoclinic	monoclinic	monoclinic	monoclinic
**Space Group**	$P\ 2_1/c$	$P\ 2_1/c$	$P\ 2_1/c$	$P\ 2_1/c$	$P\ 2_1/c$
**T [K]**	100.0(1)	100.0(1)	100.0(1)	100.0(1)	100.0(1)
**a [Å]**	12.437399(10)	12.465500(14)	12.482100(14)	12.495300(14)	12.540900(17)
**b [Å]**	16.180401(10)	16.187201(14)	16.187099(14)	16.183701(14)	16.189001(14)
**c [Å]**	33.511799(17)	33.41940(2)	33.37260(2)	33.31520(2)	33.28080(2)
**α [deg]**	90	90	90	90	90
**β [deg]**	96.7140(18)	97.0210(18)	97.191(3)	97.2820(18)	97.3460(18)
**γ [deg]**	90	90	90	90	90
**V [Å^{-3}]**	6697.74(3)	6692.85(3)	6689.86(4)	6682.67(3)	6701.36(3)
**Z**	4	4	4	4	4
**θ range [deg]**	1.127 - 27.960	1.125 - 27.970	0.957 - 27.918	0.958 - 27.966	1.119 - 27.887
**Reflns collected**	35229	35162	35180	35160	35295
**R int**	0.083	0.097	0.096	0.103	0.096
**No. of data/restr/par**	12030/908/987	9349/908/987	9087/908/987	8690/908/987	8747/908/987
**R_1 [I>2σ(I)]**	0.0607	0.0635	0.0605	0.0532	0.0477
**wR2 [all data]**	0.1704	0.1757	0.1673	0.1486	0.1348
**GoF**	1.0437	1.1322	1.1086	1.0741	1.0585
**Largest diff. pk and hole [eÅ-3]**	1.30 & -1.06	0.96 & -0.80	0.89 & -0.84	0.65 & -0.76	0.51 & -0.61

†irradiated structures of irradiated **1.py** should have 73 H atoms, however, due to the disordered acetone/isopropoxide/isopropanol sites it is not always possible to add all H at a sensible geometry, which leads to a deficit of H in the crystallographic model.

Compound	3	3	3	3	3	3	3
**Irradiation Time [s]**	0	60	150	300	600	1200	2400
**CCDC No.**	Previously reported in reference 3.[3]	-	-	-	-	-	-
**X-ray source**	synchrotron	synchrotron	synchrotron	synchrotron	synchrotron	synchrotron	synchrotron
**wavelength λ [Å]**	0.4859	0.4859	0.4859	0.4859	0.4859	0.4859	0.4859
**Formula†**	$Ti_3O_{12}C_{27}H_{54}$	$Ti_3O_{12}C_{27}H_{54}$	$Ti_3O_{12}C_{27}H_{53.5}$	$Ti_3O_{12}C_{27}H_{53.5}$	$Ti_3O_{12}C_{27}H_{53.41}$	$Ti_3O_{12}C_{27}H_{53.40}$	$Ti_3O_{12}C_{27}H_{53.38}$
**M**	714.42	714.42	713.95	713.92	713.82	713.81	713.79
**Crystal System**	monoclinic	monoclinic	monoclinic	monoclinic	monoclinic	monoclinic	monoclinic
**Space Group**	$P\ 2_1/n$	$P\ 2_1/n$	$P\ 2_1/n$	$P\ 2_1/n$	$P\ 2_1/n$	$P\ 2_1/n$	$P\ 2_1/n$
**T [K]**	100.0(1)	100.0(1)	100.0(1)	100.0(1)	100.0(1)	100.0(1)	100.0(1)
**a [Å]**	14.14330(2)	14.30870(3)	14.33620(3)	14.35080(3)	14.36390(2)	14.37560(2)	14.38160(2)
**b [Å]**	15.569000(17)	15.50630(2)	15.48090(2)	15.47010(2)	15.45390(2)	15.45260(2)	15.44830(2)
**c [Å]**	16.15910(2)	16.18960(2)	16.19150(2)	16.19400(2)	16.19670(2)	16.20810(2)	16.21090(2)
**α [deg]**	90	90	90	90	90	90	90
**β [deg]**	97.845(3)	98.747(4)	98.899(4)	98.963(4)	99.008(3)	98.992(4)	98.945(4)
**γ [deg]**	90	90	90	90	90	90	90
**V [Å^{-3}]**	3524.88(3)	3550.29(4)	3550.24(4)	3551.30(4)	3550.97(3)	3556.22(4)	3557.79(4)
**Z**	4	4	4	4	4	4	4
**θ range [deg]**	1.228 - 27.927	1.211 - 27.860	1.208 - 27.878	1.206 - 27.932	1.205 - 27.933	1.205 - 27.952	1.205 - 27.953
**Reflns collected**	18773	18926	18941	18969	18977	19012	19016
**R int**	0.068	0.128	0.11	0.108	0.091	0.084	0.116
**No. of data/restr/param**	6608/0/379	2966/444/481	3303/360/481	3558/372/481	3864/372/481	4065/372/481	3256/444/481
**R_1 [I>2σ(I)]**	0.0393	0.0647	0.0617	0.0579	0.0531	0.0539	0.0515
**wR2 [all data]**	0.1108	0.1631	0.1695	0.1570	0.1474	0.1617	0.1514
**GoF**	1.061	1.092	1.191	1.155	1.177	1.240	1.21
**Largest diff. pk and hole [eÅ-3]**	0.81 & -0.64	0.65 & -0.87	0.63 & -0.68	0.52 & -0.54	0.50 & -0.46	0.66 & -0.49	0.56 & -0.56

†The formula given is for the asymmetric unit. The crystallographic models of the irradiated structures are deficient by half an H atom. This $H_{0.5}$ is associated with an isopropanol (OH) which is not located in the structure. Formula deviations from $H_{53.5}$ are due to occupation of the acetone fragment refining to slightly greater than 0.5.

References

1. Morcombe, C. R.; Zilm, K. W., Chemical shift referencing in MAS solid state NMR. *J. Magn. Reson.* **2003,** *162* (2), 479-486.
2. Krämer, T.; Tuna, F.; Pike, S. D., Photo-redox reactivity of titanium-oxo clusters: mechanistic insight into a two-electron intramolecular process, and structural characterisation of mixed-valent Ti(iii)/Ti(iv) products. *Chem. Sci.* **2019,** *10* (28), 6886-6898.
3. Keum, Y.; Park, S.; Chen, Y.-P.; Park, J., Titanium-Carboxylate Metal-Organic Framework Based on an Unprecedented Ti-Oxo Chain Cluster. *Angew. Chem. Int. Ed.* **2018,** *57* (45), 14852-14856.
4. Winter, G.; Beilsten-Edmands, J.; Devenish, N.; Gerstel, M.; Gildea, R. J.; McDonagh, D.; Pascal, E.; Waterman, D. G.; Williams, B. H.; Evans, G., DIALS as a toolkit. *Protein Sci.* **2022,** *31* (1), 232-250.
5. Palatinus, L.; Chapuis, G., SUPERFLIP - a computer program for the solution of crystal structures by charge flipping in arbitrary dimensions. *J. Appl. Crystallogr.* **2007,** *40* (4), 786-790.
6. Betteridge, P. W.; Carruthers, J. R.; Cooper, R. I.; Prout, K.; Watkin, D. J., CRYSTALS version 12: software for guided crystal structure analysis. *J. Appl. Crystallogr.* **2003,** *36* (6), 1487.
7. Sheldrick, G. M., A short history of SHELX. *Acta Cryst. A* **2008,** *64* (1), 112-122.
8. Brown, S. E.; Mantaloufa, I.; Andrews, R. T.; Barnes, T. J.; Lees, M. R.; De Proft, F.; Cunha, A. V.; Pike, S. D., Photoactivation of titanium-oxo cluster [$Ti_6O_6(OR)_6(O_2C^tBu)_6$]: mechanism, photoactivated structures, and onward reactivity with O_2 to a peroxide complex. *Chem. Sci.* **2023,** *14*, 675-683.
9. Brown, I. D., Bond valence parameters https://www.iucr.org/resources/data/datasets/bond-valence-parameters. IUCr, Ed. 2020.
10. Spackman, P. R.; Turner, M. J.; McKinnon, J. J.; Wolff, S. K.; Grimwood, D. J.; Jayatilaka, D.; Spackman, M. A., CrystalExplorer: a program for Hirshfeld surface analysis, visualization and quantitative analysis of molecular crystals. *J. Appl. Crystallogr.* **2021,** *54* (3), 1006-1011.
11. Morra, E.; Giamello, E.; Chiesa, M., EPR approaches to heterogeneous catalysis. The chemistry of titanium in heterogeneous catalysts and photocatalysts. *J. Magn. Reson.* **2017,** *280*, 89-102.
12. Faucitano, A.; Buttafava, A.; Martinotti, F., Reactive intermediates of metal alkoxides II—an EPR investigation on irradiated titanium alkoxides. *Radiat. Phys. Chem.* **1995,** *45* (1), 31-36.
13. Guerrero, G.; Mehring, M.; Hubert Mutin, P.; Dahan, F.; Vioux, A., Syntheses and single-crystal structures of novel soluble phosphonato- and phosphinato-bridged titanium oxo alkoxides. *J. Chem. Soc., Dalton Trans.* **1999,** (10), 1537-1538.
14. Huang, Y.; Zou, G.-D.; Li, H.-M.; Cui, Y.; Fan, Y., A photoactive {Ti16} metal–organic capsule: structural, photoelectrochemical and photocatalytic properties. *New J. Chem.* **2018,** *42* (17), 14079-14082.
15. Hashizume, D.; Ohashi, Y., Photoisomerization of (2-cyanoethyl)(isonicotinic acid)cobaloxime complex in a series of isostructural host–guest complex crystals. *J. Chem. Soc., Perkin Trans. 2* **1999,** (8), 1689-1694.
